# Bilirubin-Induced Transcriptomic Imprinting in Neonatal Hyperbilirubinemia

**DOI:** 10.3390/biology12060834

**Published:** 2023-06-08

**Authors:** John Paul Llido, Emanuela Fioriti, Devis Pascut, Mauro Giuffrè, Cristina Bottin, Fabrizio Zanconati, Claudio Tiribelli, Silvia Gazzin

**Affiliations:** 1 Liver Brain Unit “Rita Moretti”, Fondazione Italiana Fegato-Onlus, Bldg. Q, AREA Science Park, 34149 Basovizza, Italy; johnpaul.llido@fegato.it (J.P.L.); emanuela.fioriti@fegato.it (E.F.); ctliver@fegato.it (C.T.); 2 Department of Science and Technology, Philippine Council for Health Research and Development, Bicutan, Taguig City 1631, Philippines; 3 Department of Life Sciences, University of Trieste, 34139 Trieste, Italy; 4 Liver Cancer Unit, Fondazione Italiana Fegato-Onlus, Bldg. Q, AREA Science Park, 34149 Basovizza, Italy; devis.pascut@fegato.it; 5 Department of Medical, Surgical and Health Sciences, University of Trieste, 34149 Trieste, Italy; mauro.giuffre@fegato.it (M.G.); cbottin@units.it (C.B.); fabrizio.zanconati@fmc.units.it (F.Z.); 6 Department of Internal Medicine, Yale School of Medicine, Yale University, New Haven, CT 06510, USA

**Keywords:** kernicterus, brain development, motor disabilities, neurologic syndrome, corplot, gene clustering, opisthotonus, schizophrenia, histone acetylation

## Abstract

**Simple Summary:**

Severe neonatal hyperbilirubinemia may damage the brain, leading to motor, cognitive, and auditory abnormalities. We recently discovered that bilirubin might act by controlling the genetic developmental program of the cerebellum, a region of the brain well known to be susceptible to bilirubin-induced damage. In this paper, we expand the study of the potential impact of bilirubin in the control of postnatal brain development to brain regions better correlating with human symptoms. The maximal abnormalities of structure and cell shape (histology) were detected 9 days after birth, fully recovering later on. Differently, the analysis of the gene expression revealed transient alterations (early after birth, then recovering) in the hippocampus (memory, learning, and cognition) and inferior colliculi (auditory functions), but permanent (until adulthood) changes in the areas of the brain involved in the control of movements, information confirmed by the abnormal results on the behavioral tests. These new findings are well in agreement with the clinic and open a way for better deciphering the neurotoxic features of bilirubin neurotoxicity and potential therapeutic approaches.

**Abstract:**

Recent findings indicated aberrant epigenetic control of the central nervous system (CNS) development in hyperbilirubinemic Gunn rats as an additional cause of cerebellar hypoplasia, the landmark of bilirubin neurotoxicity in rodents. Because the symptoms in severely hyperbilirubinemic human neonates suggest other regions as privileged targets of bilirubin neurotoxicity, we expanded the study of the potential impact of bilirubin on the control of postnatal brain development to regions correlating with human symptoms. Histology, transcriptomic, gene correlation, and behavioral studies were performed. The histology revealed widespread perturbation 9 days after birth, restoring in adulthood. At the genetic level, regional differences were noticed. Bilirubin affected synaptogenesis, repair, differentiation, energy, extracellular matrix development, etc., with transient alterations in the hippocampus (memory, learning, and cognition) and inferior colliculi (auditory functions) but permanent changes in the parietal cortex. Behavioral tests confirmed the presence of a permanent motor disability. The data correlate well both with the clinic description of neonatal bilirubin-induced neurotoxicity, as well as with the neurologic syndromes reported in adults that suffered neonatal hyperbilirubinemia. The results pave the way for better deciphering the neurotoxic features of bilirubin and evaluating deeply the efficacy of new therapeutic approaches against the acute and long-lasting sequels of bilirubin neurotoxicity.

## 1. Introduction

Severe neonatal hyperbilirubinemia, if not promptly diagnosed and managed, may account for neurological deficits recapped by the terms acute brain encephalopathy (ABE) and chronic bilirubin encephalopathy (CBE or kernicterus) [1,2,3,4]. Major neurological impairment due to bilirubin neurotoxicity in infants is still reported both in low- and middle-income countries and in Western Europe and North America [5,6,7,8].

The understanding of the molecular targets of bilirubin toxicity to the CNS is still growing, and recent is the discovery that bilirubin interferes with the epigenetic control of cerebellum (Cll) development in hyperbilirubinemic Gunn rats [9,10], potentially contributing to the Cll hypoplasia [11]. While Cll hypoplasia is the landmark of bilirubin neurotoxicity in rodent models of the disease [11,12,13,14,15], in severely hyperbilirubinemic neonates, the symptoms suggest more widespread CNS damage, involving the auditory pathway, learning and memory, and movement control [3,4,16,17,18,19,20,21]. In good agreement with the clinical scenario, the challenge with toxic amounts of bilirubin in rat organotypic brain cultures obtained from the hippocampus, inferior and superior colliculi, as well as the frontal cerebral cortex, revealed that the hippocampus (learning and memory), inferior colliculi (auditory pathway), and cerebral cortex (movement control) are even more damaged than the Cll [22]. 

To understand if a similar pattern might be present in vivo, we expanded our previous data [11] by studying the potential impact of bilirubin on the postnatal brain development of non-cerebellar regions of the Gunn rat closer to the clinical symptoms of the disease in children. To achieve the goal, we followed from birth (P2, P—postnatal age in days) to adult age the histological appearance of the developing hippocampus, inferior colliculi, and cerebral cortex of the hyperbilirubinemic (jj) animals, compared to the age-matched normobilirubinemic littermates (Ctrls). Histology was used to follow the macroscopic impact of bilirubin on CNS development, and RTqPCR was used to assess the expression of selected genes involved in the postnatal development (synaptogenesis, differentiation, repair, energy, extracellular matrix, etc.) of the investigated regions and known to be epigenetically modulated by bilirubin [11]. Finally, we performed behavioral tests to assess the potential biological relevance of the histological and genetic alterations noticed. 

## 2. Materials and Methods

### 2.1. Animals

Gunn rats (Hds Blue: Gunn-UDPGTj) [9,10], with a spontaneous mutation in the UDP glucuronosyl transferase 1a1, the enzyme responsible for bilirubin conjugation and clearance, present hyperbilirubinemia soon after birth, leading to a neurological sequel similar to the human condition [10,12,23,24,25,26]. Animals at two (P2 ± 1), nine (P9 ± 1), and seventeen (P17 ± 1) days after birth (P—postnatal age in days), and adults (from P45 to 1 year old) were obtained from the SPF animal facility of the University of Trieste (AREA Science Park, Basovizza). Ages were selected based on previous evidence [11,12,27]. Animals were housed in a temperature-controlled environment (22 ± 2 °C) and on a 12-h light/dark schedule, with ad libitum access to food and water. Based on our experimental experience demonstrating that sex is not relevant for the model, the whole litter, composed of both male and female homozygous mutated hyperbilirubinemic (jj) and normobilirubinemic (Ctrls) animals, was used. Animals were sacrificed by decapitation under deep anesthesia (tiletamina + zolazepam, 35 mg/kg, i.p.). The study was approved by the animal care and use committee of the University of Trieste (OPBA: Organismo Per il Benessere Animale) and the competent Italian Ministry (1024/2020-PR and NO2134GAZ20). All the procedures were performed according to the Italian Law (D.Lgs.26/2014) and the European Community Directive (2010/63/EU). A maximal effort was made to minimize the number of animals used and their suffering (3R rule). 

### 2.2. Hematoxylin-Eosin Stain and Morphometric Analysis

Histology was performed as previously described [11]. Immediately after sacrifice, the brains were collected and fixed in a 4% formalin buffered solution (4% formaldehyde, 37%, 33 nM NaH_2_PO_4_, 46 mM Na_2_HPO_4_), then embedded in paraffin. Sagittal sections (ML 1.1 to 1.5, stereotaxic coordinate in agreement with the interactive online Paxinos rat brain atlas http://labs.gaidi.ca/rat-brain-atlas/?ml=1.1&ap=-3&dv; accessed on 4 April 2023) of 3–5μm of thickness were obtained by a microtome (Microm-hm 340e-BioOptica, Milan, Italy), affixed on the glass slides, and dried at 60 °C for 1 h. Hematoxylin and eosin staining was performed by a Leica ST5020 Multistainer (Leica Microsystem, Milan, Italy). Staining was performed as follows: xylol 2 × 5 min; ethanol 2 × 4 min; H_2_O 1 × 3 min; hematoxylin 12 min; H_2_O 2 × 6 min; eosin 1 × 1.30 min; ethanol 2 × 3 min; xylol 1 × 3 min plus 1 × 2 min. Images were collected and analyzed with a D-Sight plus image digital microscope and scanner (Menarini Diagnostics, Firenze, Italy) by two pathologists blinded to the experimental design. The number of cells was counted in the frontal cortex (f-Ctx), parietal cortex (h-Ctx), and inferior colliculi (IC). Counting the cells in the hippocampus (Hip) was not performed due to the striking alterations at P9 and partly at P17, limiting the correct identification of the cells. The tissue thickness was quantified only in the h-Ctx region, thanks to the presence of recognizable anatomical references. For IC and f-Ctx, this was not possible due to the absence of clear boundaries or anatomical references for the structures. Three animals from each genotype and postnatal age were used. For details on the fields analyzed in each brain area, see Figure S1. The histologic study was performed at the pathological anatomy unit, Department of Medical, Surgical, and Health Sciences, University of Trieste.

### 2.3. GO Analysis, Selection of the Genes, and RT-qPCR

Based on our background on the disease (see Section 1 introduction) and histological observations, the hippocampus (Hip), frontal cortex (f-Ctx), parietal cortex (h-Ctx), and inferior colliculi (IC) were dissected from freshly collected brains and stored at −80 °C until use.

This work follows the one done by Vianello et al., in which we identified 1884 genes epigenetically modulated in the cerebellum of the hyperbilirubinemic Gunn rat. A total of 45% of them were involved in brain development, a result that we found extremely interesting [11]. To expand the study of the impact of bilirubin on the postnatal development of the four regions of interest here analyzed (f-Ctx, h-Ctx, IC, and Hip), we applied the following strategy for selecting a reasonable number of genes to study: (A) Because the brain is a heterogeneous organ where different cell types are present and this may affect gene expression, we performed literature research to ensure we chose genes relevant to the four areas of interest. Papers were searched on PubMed and Google Scholar by using as key words “f-Ctx or h-Ctx or cerebral cortex”, “Hip”, “IC”, “post-natal development”, and “rat”. Among the papers we found, we used the ones where sequencing and transcriptomic data were available, or at least a panel of genes was assessed, to guarantee the largest dataset possible [28,29,30,31,32,33,34,35,36,37,38,39,40,41]. Notably, the literature on postnatal brain maturation is largely less extensive than the papers studying fetal CNS development. (B) The list of genes that we found in the literature was then manually compared to the 1884 genes belonging to our ChipSeq, founding 213 genes. (C) To further select them, we assessed the enrichment for biological function using the available software online (http://geneontology.org/; accessed on 3 February 2021), as previously described [11]. The most represented biological functions (BF) were synaptogenesis, migration, extracellular matrix formation, differentiation, etc. (D) Finally, we selected the panel of 18 genes to study by choosing a few genes from each of the BFs in order to represent the most relevant processes of postnatal brain development. Priority was given to processes (e.g., synaptogenesis, differentiation) already known in the field when this information was available. 

The expression of the selected genes was assessed in each brain region per postnatal age by real-time PCR (RT-qPCR), as previously described [11]. Total RNA was extracted using Eurogold RNA Pure reagent (Euroclone, Milan, Italy) and retrotranscribed with the High-Capacity cDNA Reverse Transcription Kit (Applied Biosystems, Monza, Italy) according to the manufacturer’s instructions. The reaction was run in a thermal cycler (Gene Amp PCR System 2400, Perkin-Elmer, Boston, MA, USA) at 25 °C for 5 min, 37 °C for 120 min, and 85 °C for 5 min. The final cDNA was stored at −20 °C until use. Primers were designed using the Beacon Designer 8.1 software (Premier Biosoft International, Palo Alto, CA, USA) on rat sequences available in GenBank (see Table S1). A careful set-up of the amplification conditions (cDNA quantity per gene primer pair, primer concentration, and amplification protocol) was conducted to optimize the analysis in samples belonging to different postnatal ages (P2 to adult), where important physiological changes in the mRNA expression were expected. A melting curve analysis was performed to assess product specificity. At the end of the set-up, samples were analyzed in an iCycler iQ5 thermocycler (Bio-Rad Laboratories, Hercules, CA, USA). PCR was performed by mixing 250 nM each of the gene-specific sense and anti-sense primer pairs, 25 ng of the cDNA template (except for *Bmp5*, *Camlg*, *Hyal4*, *Ndufs7*, *Pfkfb1*, *Slc39a12*, and *Tnr,* which needed 5 ng of DNA; *Cacna2d4*, which needed 2 ng; and *Cacng8*, *Ndufb8*, *Ntsr1*, *Ptn*, and *Scg2* that required 1 ng of cDNA), and 1xSsoAdvance^TM^ SYBR^®^ Green Supermix (Bio-Rad Laboratories, Hercules, CA, USA). The amplification protocol used was as follows: initial denaturation at 95 °C for 30 s, followed by 40 cycles of amplification (denaturation at 95 °C for 5 s, annealing at 60 °C for 20 s, and extension at 72 °C for 30 s), and final extension at 95 °C for one minute. A different protocol was used for *Cacna2d4*, *Casp6*, *Ntsr1*, and *Ptn*: initial denaturation at 95 °C for 30 s, followed by 40 cycles of amplification (denaturation at 95 °C for 5 s and annealing at 60 °C for 20 s), and final extension at 95 °C for one minute. The relative quantification was made using the iCycler iQ Software, version 3.1 (Bio-Rad Laboratories, Hercules, CA, USA) by the Pfaffl modification of the ΔΔCT equation, taking into account the efficiencies of the individual genes. The results were normalized to the housekeeping genes and the level of mRNA was expressed relative to a reference sample [42,43,44]. The data are representative of at least three animals of each postnatal age and genotype.

### 2.4. Correlation and Clustering Studies

To identify the presence of similar trends of expression during the postnatal development of the four regions, we performed correlation studies. In control animals (physiological trend), we tested the presence of genes sharing similar trends inside each region by performing a hierarchical clustering analysis. Correlations between variables were investigated using the Spearman rank correlation test (considering the low sample size). Correlation matrices were generated with R 4.2.2 [45] by using the Hmisc (version 5.0-1 [46,47]), Performance Analytics (version 2.0.4 [48]), and Corrplot (version 0.92 [49,50]) packages. Then, a heatmap evaluation was used to highlight similarities among regions. The heatmap with the pseudocolor scale underneath the differentially expressed genes was generated using Mev 4.9.0 software [51]. Unsupervised hierarchical clustering was used to order samples and genes. The sample tree with optimized leaf ordering was drawn by using Euclidean distances and average linkages for cluster-to-cluster distance. The hierarchical clustering analysis of the 18 genes along the postnatal development was then repeated in jj rats to assess the perturbations induced by bilirubin. For all analyses, two-sided statistical significance was defined as *p* < 0.05. 

### 2.5. Behavioral Tests

To evaluate the potential behavioral abnormalities possibly linked to the altered morphometry/transcriptome data, we selected a panel of tests optimized for assessing the behavior of young to adult rats [52,53,54,55,56]. Behavioral tests assessing the instinct of correcting the position of the body relative to the position of the head were used at P9 (righting reflex) and P11/P17 (negative geotaxis). For the righting reflex, the pups were gently placed on their backs, and the time required by the animal to rotate and restore all its paws to the floor was recorded. For negative geotaxis, the pups were placed in the head downward position on an inclined plane (30° for P11, 45° for P17), and the time required for reorienting themselves towards an upward position was recorded. Rotarod and beam-walking tests were used in older (more than P45) animals. In the rotarod test, animals were placed on a horizontal rotating rod, and the time spent walking on the cylinder before falling was recorded. In the beam walking test, rats were placed on a wood beam (width 3 cm × length 100 cm, placed 30 cm from the table’s surface), and the distance/seconds the animals walked on the beam were recorded. The speed (as a function of cm/second) was then calculated. In accordance with the authorization, tests were repeated no more than twice a day, with a recovery time between the two repetitions. For rotarod and beam walking, animals were taught to perform the tests by repeating the procedure for three consecutive days. Data were collected on day 3. For all the tests, the data represent at least three animals at each postnatal age and genotype.

### 2.6. Statistical Analysis

The data were analyzed using GraphPad InStat for Windows (GraphPad Software 3.1, Inc., La Jolla, CA, USA). The Shapiro–Wilk normality test was used to study the data distribution. Differences among variables following a parametric distribution were assessed by a Student *t*-test, whereas those following a non-parametric distribution were assessed with a Mann–Whitney U test. For all analyses, two-sided statistical significance was defined as *p* < 0.05. Data are expressed as medians and interquartile ranges (quartile 1 and quartile 3) of at least 3 independent repetitions. 

## 3. Results

### 3.1. Histological Features of the Gunn Rat Brain during Postnatal Development

**Figure 1:**
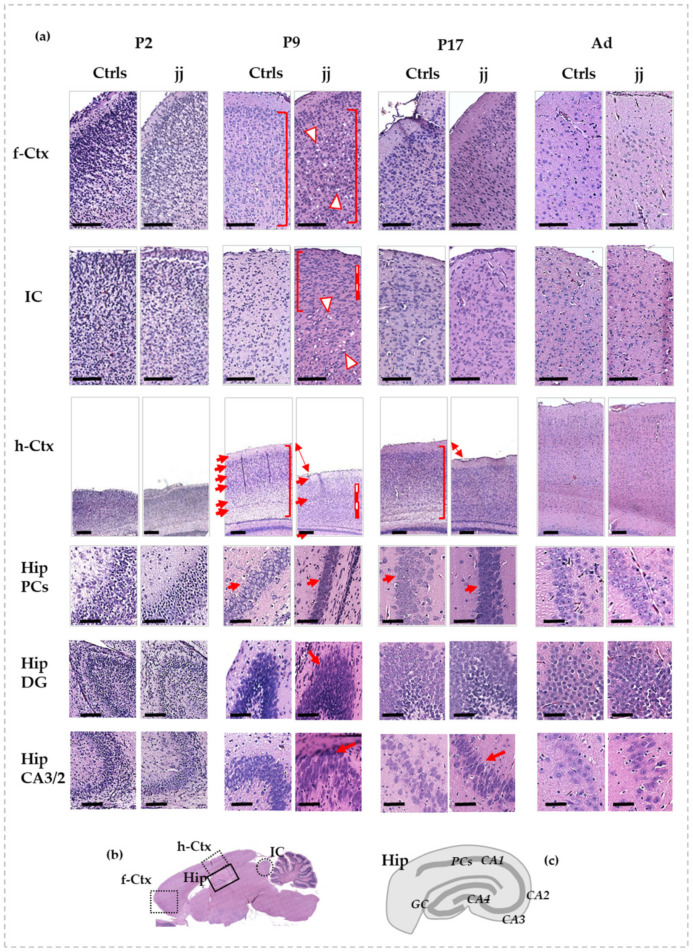
Histological features of f-Ctx, h-Ctx, IC, and Hip of developing Gunn rats compared to age-matched controls. In (**a**): Histologic pictures showing the major alterations observed in jj vs. Ctrls rats. P—postnatal age in days; Ad—adults, more than 6-month-old; jj—hyperbilirubinemic Gunn rats; Ctrls—normobilirubinemic age-matched Gunn rats; f-Ctx—frontal cortex; h-Ctx—parietal cortex; IC—Inferior colliculi; Hip—hippocampus; PCs—pyramidal cells; DG—Dentate gyrus; CA—Cornus of Ammonis. Red-white triangles: necrotic lesions. Red and white lines: attracting attention to cellular density. Red square bracket: attracting attention to cellular heterogeneity. Red arrows: drawing attention to different tissue organization, layers, architecture, and shapes of cells. Double arrow: attracting attention to the different thickness. Scale bar: 100 um in each figure. (**b**) A picture showing where each region under study is located on the rat brain. (**c**) A representative picture of the Hip structure.

Shortly after birth (P2), no histological differences between jj and Ctrls pups were noticeable in all studied regions (Figure 1a, P2, for details, and 1b for understanding where each region under study is located in the rat brain). 

At P9, differences between jj and Ctrls pups were noticeable in all the evaluated regions. In the frontal cortex (Figure 1a, f-Ctx, P9) of jj pups, despite a conserved architecture, we noticed a reduced cellular differentiation (similar shape and dimension, no distinction between nuclei, cytoplasm, and membrane—see red square brackets) and a reduced fibrillary component. Microgliosis (small dark cells) and lesions due to necrotic processes (see red-white triangles) were present. A similar scenario was observed in IC (Figure 1a, P9, IC), where the necrotic foci were limited to the core region of the structure, while the cells in the peripheral cortex of IC looked smaller, less differentiated (see red square bracket), and more numerous (see red and white line) in respect to the same region of Ctrls pups. The most striking manifestation of bilirubin neurotoxicity was observed in the parietal cortex (h-Ctx) and in the hippocampus (Hip). There was a clear reduction of the thickness of h-Ctx in jj pups vs. age-matched Ctrls (Figure 1a, P9, h-Ctx), with a reduced fibrillary component, leading to an apparent increase in cellular density (see white and red lines). Furthermore, h-Ctx in jj pups exhibited no clear distinction between cellular and molecular layers (see arrows). The heterogeneity of cell shape and orientation observable in Ctrls (see red square bracket) was also lost, indicating a reduced cellular differentiation in jj pups’ h-Ctx. On the same note, the pyramidal cells (Hip PCs, Figure 1a, P9, and Figure 1c, for an explanation of the structure of Hip) in the Cornus of Ammonis (region 1: CA1) of jj rats were undifferentiated and packed, despite the well-differentiated cells with a round shape and distinct plasma membrane and cytoplasmic compartments observable in the Ctrls, again indicating reduced cellular differentiation. Similarly, the cells in the dentate gyrus (Hip DG, Figure 1a, P9) and the CA3/2 region of the Cornus of Ammonis (Hip CA3/2, Figure 1a, P9) of jj pups appeared condensed, smaller, and with an oval, stretched shape. The presence of nuclei with compaction suggested an apoptotic process. The fibrillary component looked poorly defined and generally collapsed.

At P17 (Figure 1a, P17), both f-Ctx and IC presented few differences to age-matched Ctrls, and necrotic lesions were still not visible. The h-Ctx showed improvements despite a still-present decreased thickness and some decreased cellular differentiation. Similarly, the Hip showed improvements.

In adult animals, no differences between normo- and hyperbilirubinemic Gunn rats may be observed in any of the analyzed regions (Figure 1a, Ad).

### 3.2. Morphometric Analysis: Cell Counting and h-Ctx Thickness Quantification

To verify if the observed apparent increase in cellular density was real or due to the reduced extracellular matrix (ECM, molecular component) that was similarly noticed, we counted the number of cells in IC, f-Ctx, and h-Ctx. 

**Figure 2:**
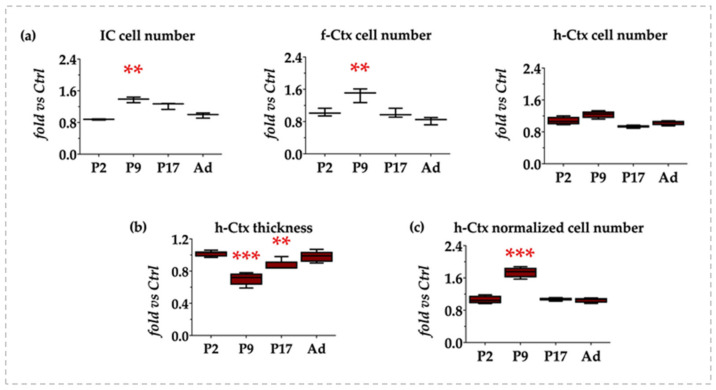
Quantification of the number of cells and h-Ctx thickness in the developing jj Gunn rats. IC—Inferior colliculi; f-Ctx—frontal cortex; h-Ctx—parietal cortex; P—postnatal age in days; Ad—adults, more than 6 months old. (**a**) Quantification of the cell number in IC, f-Ctx, and h-Ctx. (**b**) Quantification of the h-Ctx thickness. (**c**) Number of cells in h-Ctx after normalization for the thickness of the region at each postnatal age. Results are expressed as fold vs. age-matched Ctrls. Statistical significance: ** *p* < 0.01; *** *p* < 0.001.

As depicted in Figure 2a, both in IC and f-Ctx, a statistically relevant increase in the number of cells was present at P9 (both *p* < 0.01), which normalized at later postnatal ages. On the other hand, no significant difference in the absolute number of cells was quantified in the h-Ctx at any age.

We quantified the thickness of the h-Ctx region (Figure 2b) because of its evident reduction as described histologically. The reduction reached its maximal level at P9 (about 30%, *p* < 0.001 vs. age-matched Ctrls), decreasing at 13% (*p* < 0.01) at P17, and fully normalizing to the values observed in Ctrls in adult age.

When the number of cells in the h-Ctx was normalized for the thickness of the region at the same age, we obtained a 1.8-fold increase at P9 (*p* < 0.001), while no differences were present at P2, P17, or adult animals (Figure 2c). This supported the idea that the increased cell density observed in h-Ctx photomicrographs was only apparent and potentially due to the reduction of the extracellular matrix component. 

Altogether, the histology data indicated an early (P9) effect of bilirubin, with the capacity of the brain to recover later on.

### 3.3. Gene Ontology and RT-qPCR of Selected Genes

**Figure 3:**
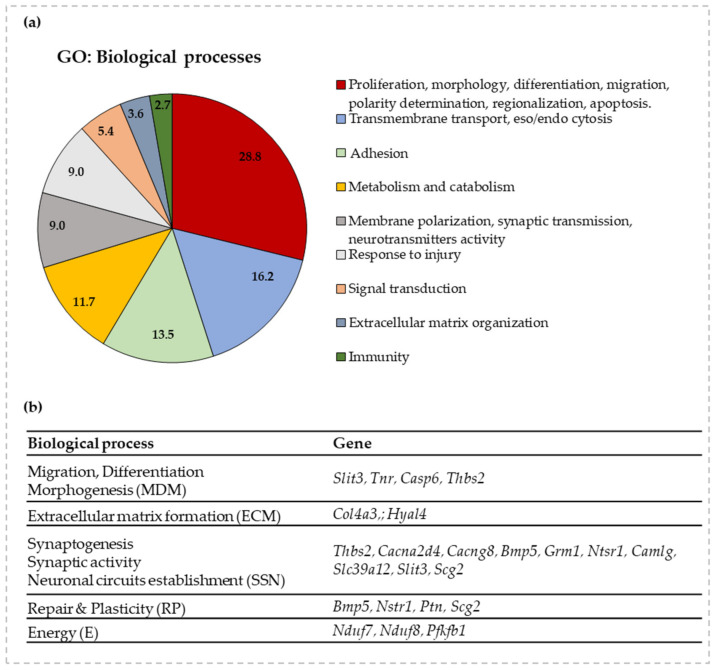
GO enrichment for biological functions and selected genes to follow the impact of bilirubin on postnatal brain development. (**a**) A graphical representation of the GO analysis. The % of genes belonging to each biological function is detailed in the pie-chart. (**b**) A list of the selected genes for each relevant biological process (with acronyms for each of them) of postnatal brain development based on GO enrichment analysis. *Slit3*—slit guidance ligand 3; *Tnr*—tenascin R, *Casp6*—caspase 6; *Thbs2*—thrombospondin-2; *Col4a3*—collagen 4a3; *Hyal4*—hyaluronidase 4; *Cacna2d4*—calcium channel, voltage-dependent, α 2/Δ subunit 4; *Cacng8*—calcium voltage-gated channel auxiliary subunit gamma 8; *Bmp5*—bone morphogenetic protein 5; *Grm1*—glutamate metabotropic receptor 1; *Ntsr1*—neurotensin receptor 1; *Camlg*—calcium modulating ligand; *Scg2*—secretogranin II; *Ptn*—pleiotrophin; *Nduf7/8*—NADH: ubiquinone oxidoreductase core subunit 7/8; *Pfkfb1*—6-phosphofructo-2-kinase/fructose-2,6-biphosphatase 1; *Slc39a12*—solute carrier family 39 member 12.

Because the histology confirmed the possible effect of bilirubin on processes involved in brain development, we moved forward by investigating a panel of genes involved in brain maturation previously reported to be modulated by bilirubin [11]. As a result of the selection procedure described in Methods, a panel of 18 genes was chosen to follow the most relevant processes of postnatal brain development (Figure 3). Priority was given to genes known in the literature concerning bilirubin neurotoxicity when available [2,25,57]. 

### 3.4. RTqPCR Results in Ctrls (Physiologic Expression)

**Figure 4:**
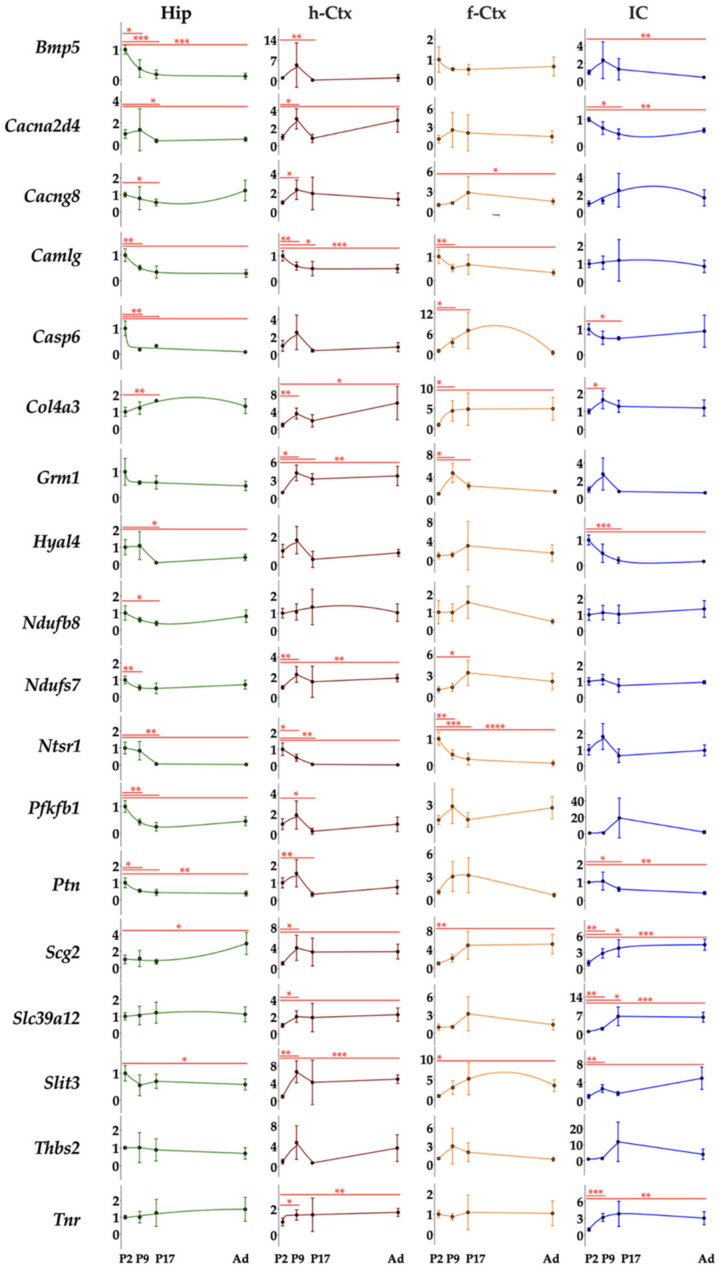
Region specific mRNA transcription level during the postnatal development of Ctrl rats. Hip—hippocampus; h-Ctx—parietal cortex; f-Ctx—frontal cortex; IC—inferior colliculi; *Bmp5*—bone morphogenetic protein; *Cacna2d4*—calcium voltage-dependent calcium channel complex alpha-2/delta subunit family; *Cacng8*—calcium voltage-gated channel auxiliary subunit gamma 8; *Camlg*—calcium modulating ligand; *Casp6*—caspase 6; *Col4a3*—collagenase 4a3; *Grm1*—glutamate metabotropic receptor 1; *Hyal4*—hyaluronic acid 4; *Nduf7/8*: NADH—ubiquinone oxidoreductase (complex I) subunit 7/8; *Ntsr1*—neurotensin receptor 1; *Pfkfb1*—6-phosphofructo-2-kinase/fructose-2,6-biphosphatase 1; *Ptn*—pleiotrophin; *Scg2*—secretogranin II; *Slc39a12*—solute carrier family 39 member 12; *Slit3*—slit guidance ligand 3; *Thbs2*—thrombospondin 2; *Tnr*—tenascin R. Data are in fold vs. P2 Ctrls. Statistical significance: * *p* < 0.05; ** *p* < 0.01; *** *p* < 0.001.

First, we assessed the expression of the 18 genes in the four regions of interest along the postnatal brain development and until the adult age of Ctrls animals (Figure 4). This step was propedeutic to understand the impact of bilirubin.

Hip (Figure 4, Hip) was the region presenting the higher number of genes with a maximal expression at P2 (11 on 18: *Camlg*, *Ntsr1*, *Bmp5*, *Casp6*, *Ptn*, *Slit3*, *Grm1*, *Pfkb1*, *Nduf7*, *Nduf8*, and *Thbs2*); *Hyal4* and *Cacng8* at P9; *Col4a3* and *Slc39a12* at P17; and *Cacng8*, *Tnr*, and *Scg2* in adult animals. 

h-Ctx (Figure 4, h-Ctx) presented only two genes with maximal expression at P2 (*Camlg and Ntsr1*), but 12 at P9 (*Bmp5*, *Casp6*, *Ptn*, *Slit3*, *Grm1*, *Pfkb1*, *Nduf7*, *Cacna2d4*, *Hyal4*, and *Thbs2*, the last presenting a second similar peak in Ad). One peak (*Nduf8*) was recorded at P17, while 3 genes *(Col4a3*, *Slc39a12*, and *Tnr*) had their peak in adult life. 

IC and f-Ctx displayed a more widespread maximal expression along brain maturation. In IC, 3 genes had the maximum at P2 (*Casp6*, *Hyal4*, *Cacna2D4*); 6 at P9 (*Ntsr1*, *Bmp5*, *Ptn*, *Grm1*, *Nduf7*, and *Col4a3*); 6 at P17 (*Camlg*, *Pfkfb1*, *Thbs2*, *Col4a3*, *Cacng8*, and *Tnr*); and 3 genes in adult life (*Slit3*, *Nduf8*, and *Scg2*). 

In f-Ctx, 4 genes had the peak at P2 (*Camlg*, *Ntsr1*, *Bmp*, and *Casp6*); 4 at P9 (*Grm1*, *Thbs2*, *Cacn2d4*, and *Pfkfb1*—the last with a second peak in Ad); 8 at P17 (*Ptn*, *Slit3*, *Nduf7*, *Nduf8*, *Hyal4*, *Slc39a12*, *Cacng8*, and *Tnr*); and *Col4a3* and *Scg2* in adults (Figure 4, f-Ctx, and IC). 

Based on this naïf analysis, Hip looked to be the first region developing (mostly at P2: 11 genes), followed by h-Ctx (mostly at P9: 12 genes), then IC and f-Ctx with a well-distributed number of peaks between P2 (3 both) and P9 (6 and 4, respectively), but a maximal transcriptional development at P17 (6 and 9 peaks, respectively) (see Figure S2).

### 3.5. Heatmap Analysis of the Gene Expression in Ctrls along the Postnatal Brain Development

**Figure 5:**
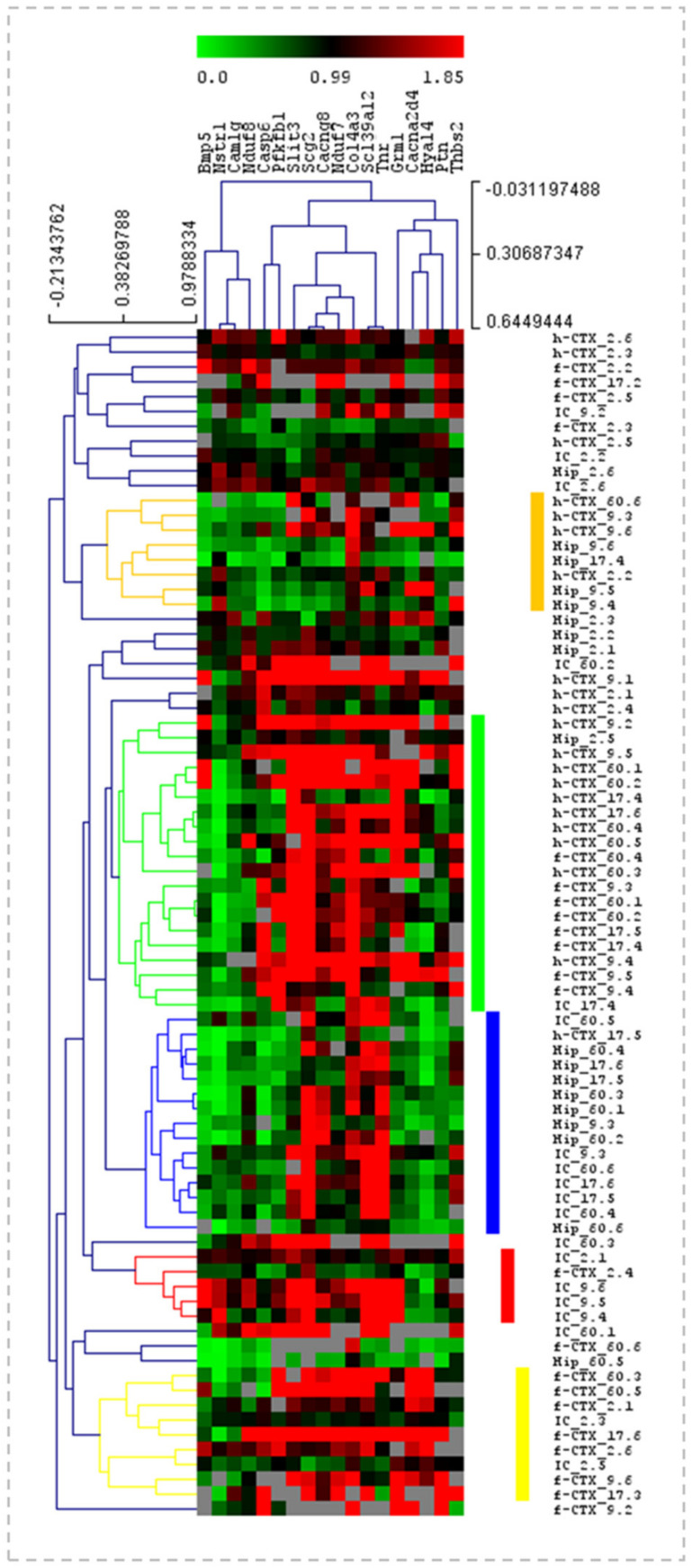
Heatmap analysis of the gene expression in Ctrls. Heatmap with the pseudocolor scale underneath, unsupervised hierarchical clustering is used to order samples and genes. The sample tree with optimized leaf-ordering is drawn using Euclidean distances and average linkages for cluster-to-cluster distance. Color scale: green = lower expression; red = higher expression. Columns: the 18 genes under analysis. Rows: the expression of each gene in the selected regions, and at a specific postnatal age. f-Ctx—frontal cortex; h-Ctx—parietal cortex; Hip—hippocampus; IC—inferior colliculi; *Bmp5*—bone morphogenetic protein; *Cacna2d4*—calcium voltage-dependent calcium channel complex α-2/Δ subunit family; *Cacng8*—calcium voltage-gated channel auxiliary subunit gamma 8; *Camlg*—calcium modulating ligand; *Casp6*—caspase 6; *Col4a3*—collagenase 4a3; *Grm1*—glutamate metabotropic receptor 1; *Hyal4*—hyaluronic acid 4; *Nduf7/8*: NADH—ubiquinone oxidoreductase (complex I) subunit 7/8; *Ntsr1*—neurotensin receptor 1; *Pfkfb1*—6-phosphofructo-2-kinase/fructose-2,6-biphosphatase 1; *Ptn*—pleiotrophin; *Scg2*—secretogranin II; *Slc39a12*—solute carrier family 39 member 12; *Slit3*—slit guidance ligand 3; *Thbs2*—thrombospondin 2; *Tnr*—tenascin R.

The analysis of the data we performed before allowed us to individuate the temporal dynamics of transcriptional development in each region. To identify genes presenting similar trends of expression among the four regions, we used a heatmap analysis (Figure 5). In heat maps, the data are represented by a color scale from green (the lower expression level) to red (the higher expression level) and are organized based on a hierarchical range of similarity (in expression), allowing visual identification of genes presenting the same trend (clusters). The major hierarchical clusters are depicted in different colors at the right of the heatmap. Little similarities in gene expression between the regions of Ctrls animals were identified. The first cluster grouped h-Ctx and Hip, the second cluster pointed out the similarities mainly between h-Ctx and f-Ctx. In the third, we noticed correlations between IC and Hip, while the last two groups mostly revealed little similarities between f-Ctx and IC.

### 3.6. RTqPCR Results on Hyperbilirubinemic Animals

When we moved to the analysis of the expression of the 18 genes in the four regions along the postnatal development of hyperbilirubinemic rats vs. Ctrls animals, we noticed a widespread effect of bilirubin in each region. Indeed, differences among the gene’s modulation and differences among regions looked to be present.

In general, if altered, *Slit3*, *Col3a4*, *Cacna2d4*, *Ptn*, *Nduf7*, and *Nduf8* expression were reduced. All the other genes presented both behaviors (up and down). 

**Figure 6:**
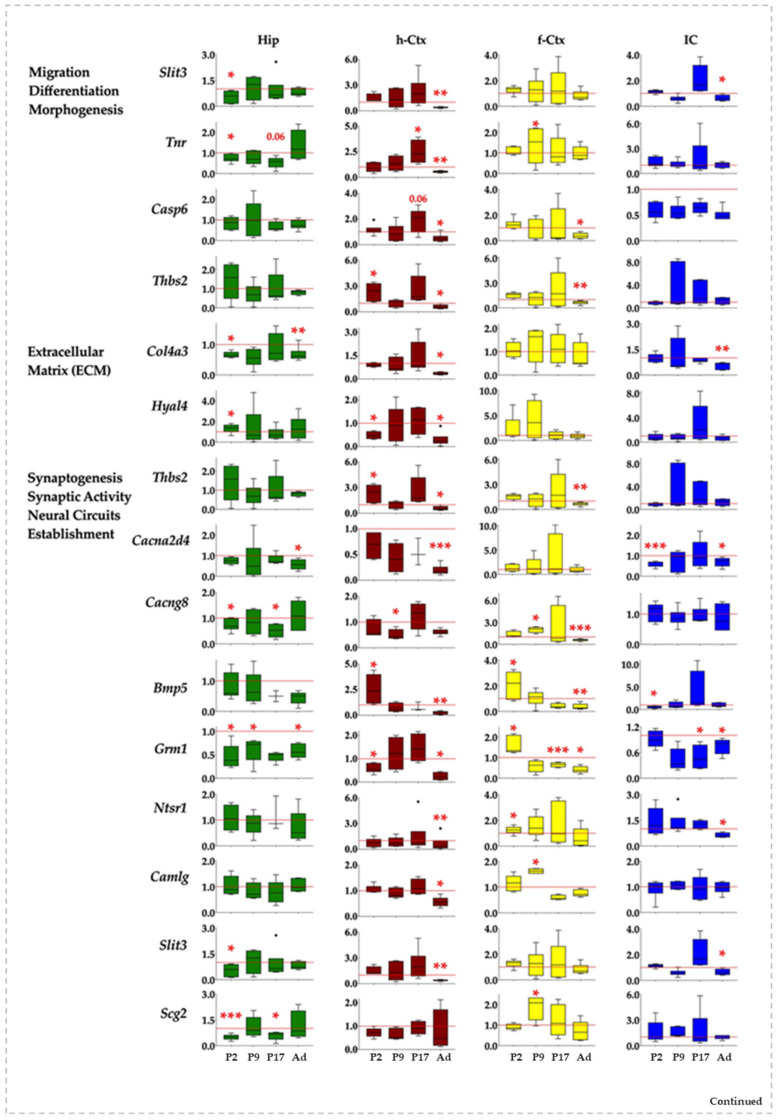

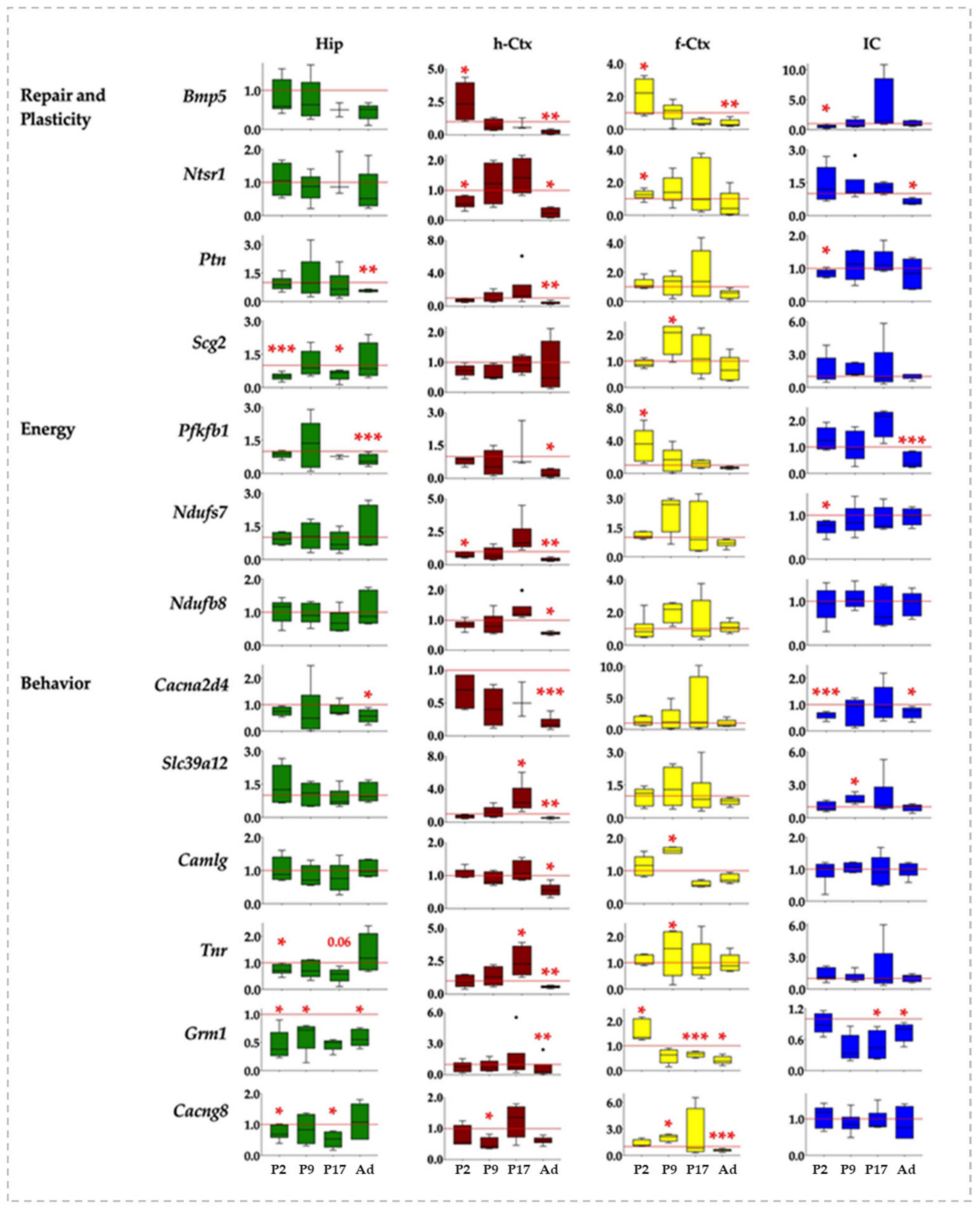
Gene and region-specific mRNA transcription levels in hyperbilirubinemic Gunn rats during postnatal development vs. age-matched Ctrl littermates. Gene expression was presented based on the GO functions plus the behavior group, which was added based on literature evidence of single gene involvement in neurological conditions/diseases. Hip—hippocampus; h-Ctx—parietal cortex; f-Ctx—frontal cortex; IC—inferior colliculi. Red line: Ctrls (=1 at each postnatal age). *Bmp5*—bone morphogenetic protein; *Camlg*—calcium modulating ligand; *Casp6*—caspase6; *Col4a3*—collagenase 4a3; *Cacna2d4*—calcium voltage-dependent calcium channel complex α-2/Δ subunit family; *Cacng8*—calcium voltage-gated channel auxiliary subunit gamma 8; *Grm1*—glutamate metabotropic receptor 1; *Hyal4*—hyaluronic acid 4; *Ntsr1*—neurotensin receptor 1; *Nduf7/8*: NADH—ubiquinone oxidoreductase (complex I) subunit 7/8; *Slit3*—slit guidance ligand 3; *Scg2*—secretogranin II; *Slc39a12*—solute carrier family 39 member 12; *Tnr*—tenascin R; *Thbs2*—thrombospondin 2; *Ptn*—pleiotrophin; *Pfkfb1*—6-phosphofructo-2-kinase/fructose-2,6-biphosphatase 1. Data are in fold vs. age-matched Ctrls. Statistical significance: * *p* < 0.05; ** *p* < 0.01; *** *p* < 0.001.

In Hip, the genes were significantly down-regulated 16 times, while only 1 significant up-regulation event was noticed (Figure 6, Hip). At *P2, Slit3*, *Col4a3*, *Cacng8*, *Grm1*, *Scg2*, and *Tnr* were reduced, while *Hyal4* accounted for the only significant up-modulation in this region. *Grm1* was the only gene significantly altered (down-regulated) 9 days after birth, with *Cacng8*, *Scg2*, and *Tnr* significantly reduced at P17 and *Col4a3*, *Cacna2d4*, *Grm1*, *Ptn*, and *Pfkfb1* significantly reduced in adult age. 

The h-Ctx accounted for the maximal number of modulatory events (total 24: 20 down and 4 up-regulations). Early after birth (P2), we noticed a statistically significant reduction of gene expression for *Hyal4*, *Ntsr1*, and *Nduf7*, while *Thbs2* and *Bmp5* were significantly enhanced. *Cacng8* was the only significant alteration (down-regulation) noticed at P9. One week later (P17), Tnr and *Slc39a12* mRNA were significantly increased, while in adults 16 genes were significantly reduced (*Slit3*, *Tnr*, *Casp6*, *Thbs2*, *Col4a3*, *Hyal4*, *Cacna2d4*, *Bmp5*, *Grm1*, *Ntsr1*, *Camlg*, *Ptn*, *Pfbkb1*, *Nduf7*, *Nduf8*, and *Slc39a12*), and none significantly increased (Figure 6, h-Ctx). 

f-Ctx presented more up-regulated (9) than down-regulated significant events (6, on a total of 15). *Bmp5*, *Grm1*, *Ntsr1,* and *Pfbkb1* at P2; *Tnr*, *Cacng8*, *Camlg,* and *Scg2* at P17 expression was increased. Since P17, we have noticed an opposite effect of bilirubin, with a significant reduced expression of *Grm1* (P17) and *Thbs2*, *Cacng8*, and *Bmp5* in adults.

Finally, in IC, we noticed a majority of significant down-regulated events (11) and only one significant increase in gene expression (Figure 6, IC). Two days after birth, *Cacna2d4*, *Bmp5*, *Ptn*, and *Nduf7* expression were reduced. The only significant up-regulation observed in the region was *Slc39a12* at P9. At P17 *Grm1*, and in adults, *Slit3*, *Col4a3*, *Cacna2d4*, *Grm1*, and *Pfkfb1* were significantly decreased.

In summary, and from a different point of view, Hip looked to be the region of the brain suffering the early effect of bilirubin (modulation, P2: 8, P9: 1; P17: 3, Ad: 5); followed by f-Ctx (P2: 4, P9: 5; P17: 1, Ad: 6); and IC (P2: 4, P9: 1; P17: 1, Ad: 6); with the h-Ctx suffering the late and persistent consequences (P2: 5, P9: 1; P17: 3, Ad: 15).

### 3.7. Correlation Analysis of Bilirubin Impact on Genes Expression

**Figure 7:**
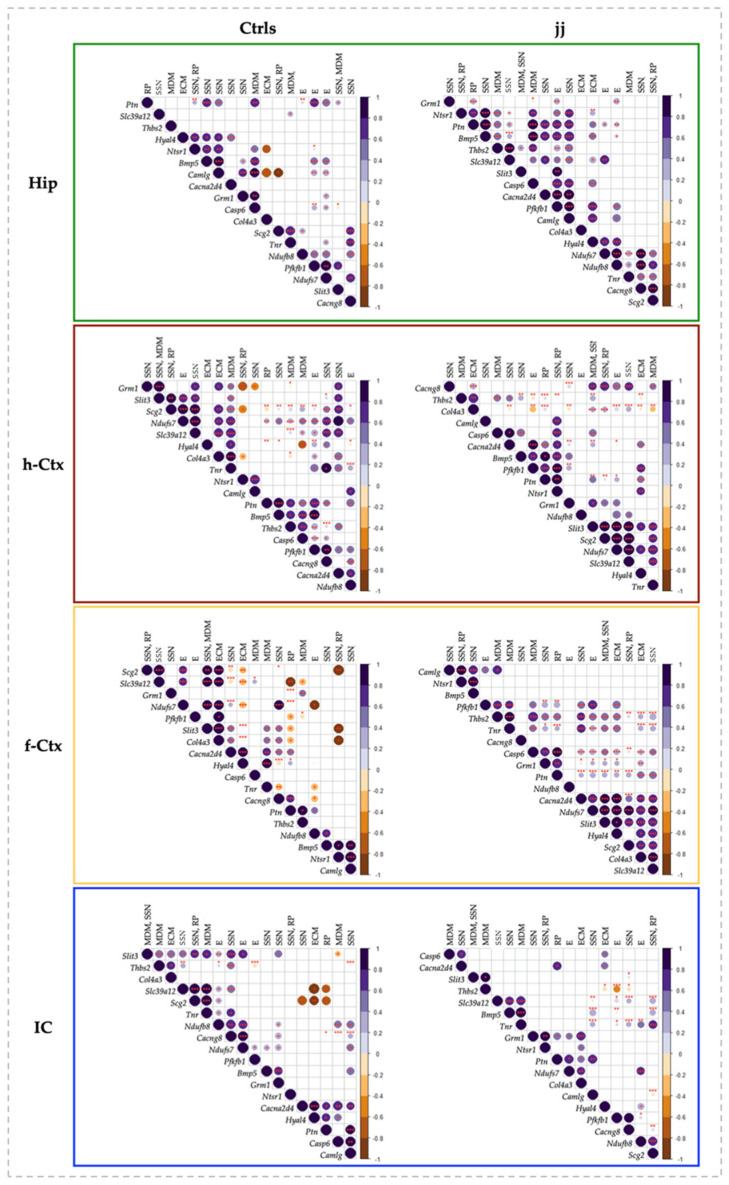
Correlation charts of studied genes in different brain regions of jj and Ctrls rats. The upper-right side shows the correlation coefficient and *p*-value for each pairwise correlation. The diameter and color depth of the dots were proportional to the R coefficient of correlation, while the color tone indicated if the correlation was positive or negative. The statistical significance of the correlation was indicated as follows: * *p* < 0.05; ** *p* < 0.01; *** *p* < 0.001. Only significant correlations were reported. The order of the genes along the X and Y-axes of each correlogram was automatically clustered depending on the R-values. The corrplots of both Ctrls and jj rats were normalized to P2 Ctrls. Hip—hippocampus; h-Ctx—parietal cortex; f-Ctx—frontal cortex; IC—inferior colliculi; *Bmp5*—bone morphogenetic protein; *Camlg*—calcium modulating ligand; *Casp6*—caspase6; *Col4a3*—collagenase 4a3; *Cacna2d4*—calcium voltage-dependent calcium channel complex α-2/Δ subunit family; *Cacng8*—calcium voltage-gated channel auxiliary subunit gamma 8; *Grm1*—glutamate metabotropic receptor 1; *Hyal4*—hyaluronic acid 4; *Ntsr1*—neurotensin receptor 1; *Nduf7/8*: NADH—ubiquinone oxidoreductase (complex I) subunit 7/8; *Slit3*—slit guidance ligand 3; *Scg2*—secretogranin II; *Slc39a12*—solute carrier family 39 member 12; *Tnr*—tenascin R; *Thbs2*—thrombospondin 2; *Ptn*—pleiotrophin; *Pfkfb1*—6-phosphofructo-2-kinase/fructose-2,6-biphosphatase 1. The upper row resumes the biological function of each gene based on GO analysis. RP—repair and plasticity; SSN—synaptogenesis, synaptic activity, and neuronal circuit establishment; MDM—migration, differentiation, and morphogenesis; ECM—extracellular matrix formation; E—energy.

To decipher the molecular data, we assessed the potential correlations between the genes in the same region. The correlation matrix was reordered according to the correlation coefficient through hierarchical clustering. Thus, genes sharing a similar pattern of expression were grouped together, indicating these genes are co-expressed. Because the clusters in Ctrls represented the physiological development leading to a perfectly functional brain, by comparing the clusters in jj rats with the clusters in Ctrls, we aimed to better understand the potential impact of bilirubin on brain development. As the striking differences in Figure 7 showed, the correlation analysis corroborated the differential impact of bilirubin among the four regions. 

In Hip of the Ctrls animal, four clusters were noticed (Figure 7, Hip, Ctrs). In the text, we added a short description of the function of the genes only the first time they were mentioned. Details and references may be found in Table S2). A positive correlation was found among *Hyal4 * (an important component of the extracellular matrix [29,33,37]), *Ntsr1* (synaptogenesis, plasticity, and neuronal circuit formation [28,58,59,60,61]), *Bmp5* (extension and survival of dendrites [29,33,39]), and *Camlg* (trafficking of post-synaptic GABA receptors [38,62]) in cluster 1. In cluster 2, we found *Grm1* (a metabotropic glutamate receptor involved in post-synaptic activity and in neurogenesis in the subventricular zone—SZV [28,38,40,62,63]), and *Casp6* (apoptosis, with relevant physiologic roles in achieving the final number of cells and tissue architecture during postnatal development [64,65,66,67]). Cluster 3 included *Scg2* (synaptogenesis, neuronal circuit formation, plasticity, and repair [38]) and Tnr (a constituent of peri-neuronal nets). Acting in both a negative/positive way on neurons and neurites growth, synapses maintenance, and oligodendrocyte differentiation; and regulating astrocyte glutamate uptake in adult brain [28,37,68,69]); while cluster 4 grouped genes involved in the cellular bio-energetic activities (*Nduf7* and *8*: part of the core subunit of the mitochondrial membrane respiratory chain NADH dehydrogenase complex I [28,70,71,72]; and *Pfkfb1*: bifunctional enzyme acting on cellular glucose homeostasis by activating glycolysis and inhibiting gluconeogenesis [28,73]). Additional significant correlations were reported in Figure 7, Hip, Ctrls. Among them, negative correlations (opposite trend of expression) were present between *Col4a3* (extracellular matrix formation [31,37]), *Camlg* and *Ntsr1* (synaptogenesis, plasticity, neuronal circuitry [28,58,59,60,61]); *Scg2* and *Camlg*; *Ptn* and *Scg2*; and *Thbs2* and *Grm1*. 

As seen by comparing the picture representing the Hip of Ctrls with the picture of the clusters in Hip of jj rats (Figure 7, Hip, jj vs. Ctrls), bilirubin increased the number of correlated genes and inverted the negative correlations present in Ctrls animals, suggesting a flattening of the transcript trends. Moreover, Ctrls cluster 1 was partly destroyed (*Hyal4* and *Camlg* missed; *Thbs2*, *Camlg*, *Ptn*, and *Slc39a12* were introduced), and clusters 3 and 4 were mixed (compare clusters 3 and 4 of Ctrls animals with cluster 4 of jj animals: *Nduf7*, *Nduf8*, *Tnr*, *Cacng8*, and *Scg2*).

h-Ctx of Ctrls animals presented 7 clusters. None of these clusters included the same genes that the clusters of Hip. *Grm1* and *Slit3* (chemo-repellent in axon guidance [37]) had in cluster 1. Cluster 2 included *Slit3*, *Slc39a12* (Zn transporter, with Zn as an important co-factor of multiple enzymes and in multiple biological functions [74,75,76,77,78,79,80]), *Scg2*, and *Nduf7*. The following 3 mini-clusters grouped *Col4a3* and *Tnr2* (cluster 3); *Ntsr1* and *Camlg* (cluster 4); while the mini-cluster 7 (at the bottom of the corplot) included *Nduf8* and *Cacna2d4* (a voltage-gated calcium channel, important in synapses formation [28,38]). The larger cluster (#5) included 5 genes: *Ptn* (repair and plasticity [41,81,82,83]), *Thbs2* (compartmentalization of the ECM, a key process in determining the correct directional migration of cells and growth of synapses [37,84,85,86]), *Bmp5*, *Casp6*, and *Pfkfb1*. *Casp6* and *Pfkfb1* were also part of a sub-cluster of cluster 6, together with *Cacng8* (promoting the trafficking of AMPA glutamate receptors to the synapse and modulating their activity [28,38]). Among the negative correlations, some were h-Ctx-specific (e.g., *Camlg* with *Grm1*; *Thbs2* with *Col4a3*), while others looked to be shared with Hip (e.g., *Col4a3* with *Ntsr1*; *Ptn* with *Scg2*). 

Concerning the effects of bilirubin, Ctrls cluster 2 was maintained in jj rats (see cluster 4 of jj h-Ctx), cluster 5 was partly destroyed (*Thbs2* and *Casp6* missed), and the Ctrls mini-clusters 1, 3, 4, 6, and 7 were destroyed. Additionally, the negative correlations suffered rearrangements. 

In the f-Ctx of Ctrls rats, we found 7 small clusters (e.g., #1: *Scg2*, *Slc39a12*; #2: *Slit3*, *Col4a3*, *Cacna2d4*, *Hyal4*; #3: *Tnr*, *Cacng8*; #4: *Cacng8*, *Ptn*; #5: *Ptn*, *Thbs2*; #6: *Nduf8*, *Bmp5*, and #7: *Bmp5* with *Ntsr1* and *Camlg*). This region presented the maximal number (#8) of negative correlations that were again region specific, with only the already observed *Col4a3* and *Ntsr1* negatively correlated genes shared with Hip. 

In hyperbilirubinemic rats, clusters #1 and 2 of Ctrls melted together (cluster 4 of jj animals) and moved to the bottom of the hierarchical clustering. Vice-versa, cluster 7 of Ctrls moved to the top of the jj hierarchical clustering. All the other clusters noticed in normobilirubinemic rats were lost. 

Finally, in IC, 8 clusters were identified (#1: *Slit3*, *Thbs2*, *Col4a3*; #2: *Slc39a12*, *Scg2*, *Tnr*, *Nduf8*; #3: *Tnr*, *Nduf8*, *Cacng8*; the partly overlapped clusters #4: *Nduf8*, *Cacng8*, *Nduf7*; #5: *Nduf7*, *Pfbkb1*; #6: *Bmp5*, *Grm1*; #7: *Cacna2d4*, *Hyal4*, *Ptn*; and #8: *Casp6* and *Camlg*). Among the negative correlations, IC shared similar trends with f-Ctx (*Cacna2d4*, Scg; *Hyal4*, *Scg2*, *Slc39a12*; and *Ptn*, *Slc39a12*), while the negative correlation between *Ptn* and *Scg2* was shared with both Hip and h-Ctx. 

Bilirubin destroyed clusters 3–8 of Ctrls animals, forming the new clusters #1 and #4–7 of jj rats. 

In summary, bilirubin induced a transcriptional di-syncronicity (cluster destruction) and a flattening (inversion of the negative correlations) of the gene expression in jj rats with respect to what was observed in normobilirubinemic subjects (physiologic trend) (Figure 7), with permanent transcriptional imprinting mainly in the h-Ctx of hyperbilirubinemic subjects (Figure 6, h-Ctx, Ad).

### 3.8. Behavioral Tests

**Figure 8:**
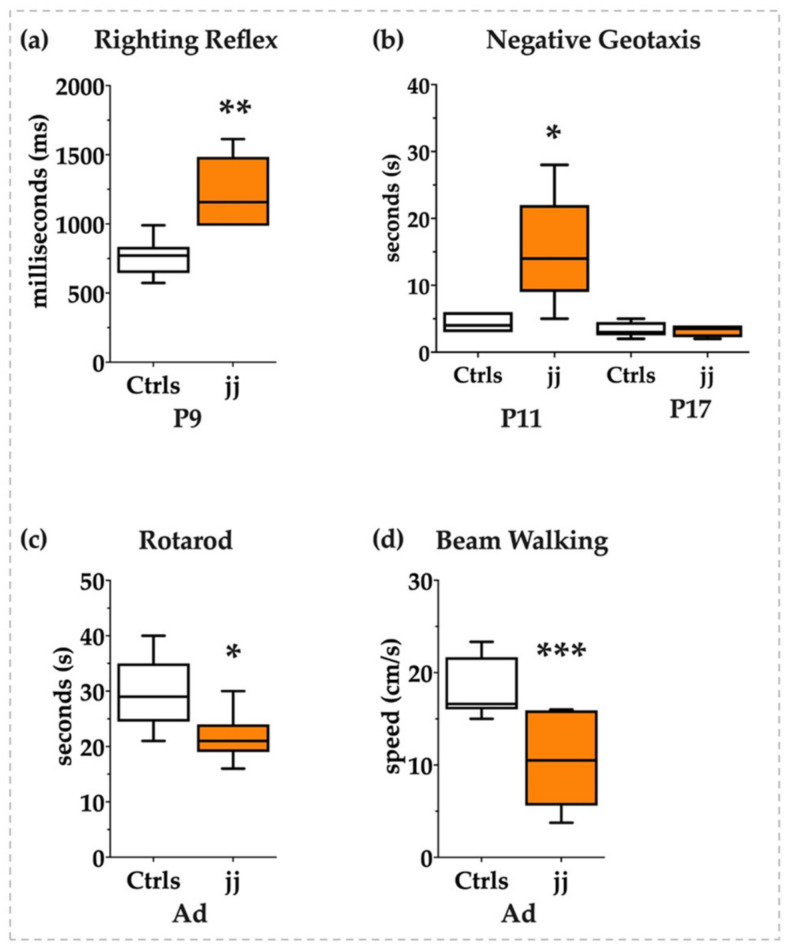
Behavioral tests. White bars: Ctrls; orange bars: hyperbilirubinemic rats. P: postnatal age in days, Ad: adults, from P45 to 1 year old. (**a**) Righting reflex and (**b**) negative geotaxis in pups. (**c**) Rotarod and (**d**) beam walking tests in adult animals. Statistical significance: * *p* < 0.05; ** *p* < 0.01; *** *p* < 0.001.

To understand if jj Gunn rats were able to recover in adult life from the early induced damage as reported by histology or if the transcriptomic modulation we noticed was nevertheless impacting the neurologic functions, we performed a panel of behavioral tests to investigate the neurological sequelae of bilirubin toxicity from the young postnatal age to adult life. As shown in Figure 8, at P9 jj Gunn pups required almost double the time to gain the position on their legs in the righting reflex tests (Figure 8a) and almost three times more to orientate the head in the uphill position in the negative geotaxis test at P11. The negative geotaxis test normalized later on, at P17 (Figure 8b). 

The normalization might be interpreted in two opposite ways: (a) as a recovery from the brain damage presenting its maximum at P9, in line with the histology, or (b) as the physiological improvement of the motor abilities that make the test too easy for the animal, thus making the test unable to adequately challenge the pups and detect the inabilities. 

We added behavioral tests investigating the acquired motor abilities in adult life to answer the question. As both the rotarod and the beam walking revealed (Figure 8c and Figure 8d, respectively), jj Gunn rats presented a long-lasting deficit of equilibrium, coordination, and movement control in agreement with the unrecovered gene expression.

Thus, behavioral tests confirmed the presence of neurologic damage.

## 4. Discussion

Severely hyperbilirubinemic neonates may present auditory, motor, and cognitive symptoms suggestive of the regions of the brain affected by bilirubin neurotoxicity. Neurological tests, imaging approaches, and extremely rare autopsy reports support the selective vulnerability of defined brain areas to bilirubin toxicity [16,21,87,88]. 

The Gunn rat, the spontaneous model of neonatal hyperbilirubinemia [9,10,12,25,89,90], is a useful model to address the biomolecular processes involved in bilirubin neurotoxicity but has been rarely used for studying extra Cll regions. In this exploratory work, we described the transcriptomic effect of bilirubin in regions of the brain of the Gunn rat more closely linked to human symptoms.

We report the presence of early and transient histologic abnormalities in all the regions, indicative of an altered development, but also an apparent recovery in adulthood. There are very few possibilities for discussing this result. While there is a large literature describing bilirubin damage to Cll in animal models of neonatal hyperbilirubinemia [10,13,24,91], the regions we studied in this work have been almost neglected, and no reports are available along the postnatal brain development [92,93]. The experimental plan we used mimics a human neonate with untreated severe hyperbilirubinemia leading to neurological sequelae. In the clinic, infants are usually treated to rapidly decrease the level of bilirubin in their blood, and neurological and MRI investigations are performed on them. The rare autopsies represent an extreme situation where it is not surprising to find neuronal death. Moreover, in a large portion of autopsy reports, both hyperbilirubinemic neonates and control infants present very important co-morbidities. Thus, the information coming from autopsies has to be taken with caution. In our work, we noticed an increase in the cell number in IC and f-Ctx at P9, with the total number of cells lately normalizing. This might be explained in different ways: (a) a temporary increase in cell proliferation. While neurogenesis is considered to be no longer present after birth (but recent data suggest this is not fully true both in animals and humans [67,94,95,96,97,98,99]), the proliferation of astrocytes, oligodendrocytes, and cells forming the vasculature of the brain is certainly still ongoing. (b) Microgliosis, which we noticed by histology, is a known feature of bilirubin neurotoxicity [25,100,101,102], and in the course of microgliosis, microglia cells are not only activated but also proliferating [103]. The same happens in astrogliosis, another well-known feature of bilirubin toxicity to the brain [57,101,104,105]. Thus, P9 might represent the nadir of the glial reaction to bilirubin toxicity. (c) A temporary reduction of apoptosis that plays a crucial physiologic role in the final definition of the cell number and architecture of the developing brain. With the precautions previously explained, in the autopsy of hyperbilirubinemic infants, death of neurons has been reported, sometimes compensated by an increased number of astrocytes [106]. A decrease in the ratio of N-acetyl-aspartate/choline and creatine has been reported in MRI studies in hyperbilirubinemic infants, and it has been interpreted as the loss of neurons or as a neuronal/axonal dysfunction [87]. Similar data have been obtained also in experimental models [107], where long-lasting electron microscopy abnormalities of cellular organelles in Hip and cerebral Ctx (and other regions we did not study in this work) have been noticed without commenting on the total number of cells [92]. As reported by Amin et al., bilirubin-induced damage may not be detected by gross histology, despite being present [88]. Thus, immunofluorescence to count the individual types of cells (e.g., neurons, astrocytes, and oligodendrocytes) will be functional to understand what we observed and to elucidate if, in adulthood, the cells recovered from the alterations observed early after birth or if a disequilibrium among the cell’s types persists.

Different are the genomic results. 

Interesting is the transcriptomic imprinting observed in the h-Ctx, a region that looks to be mostly insensitive to early modulations. Despite being highly speculative, it is suggestive to notice that the late dynamic of damage in the h-Ctx remembers the Cll, a region of the Gunn rat requiring a long time to manifest the damage due to bilirubin. In Cll, the presence of a window of susceptibility, from P6 to P10, is well-known. If the animal is exposed to phototherapy (PT, decreasing bilirubin challenging) in this window of time, Cll will develop normally; if PT is performed before or after, Cll hypoplasia will develop [26]. This phenomenon has been explained by the presence of maximally active processes of Cll development during P6–P10 [108]. It is speculative but fascinating that most of the genes permanently altered in h-Ctx present their maximal physiologic expression at P9. The hypothesis that the window of susceptibility P6–10 may also be true for h-Ctx has to be experimentally verified. The rule is partly confirmed by expanding the reasoning to the other genes permanently altered in the other regions (50% in f-Ctx; 20% in Hip; 50% in IC). From a different point of view, in these three regions, mostly characterized by early and transient transcriptomic alterations, the maximal part of the genes has a physiological peak of expression before or after P9. In any case, even if the hypothesis of the window of susceptibility looks attractive for the h-Ctx, it cannot be considered a general rule. Altogether, the data confirm a different susceptibility to damage among the regions, a different sensitivity that is not due to the amount of bilirubin, which is equal in all regions [25]. Further studies are needed. 

Bilirubin neurotoxicity is known to play out through multiple mechanisms [25,109]. Its effect on glutamatergic neurons has often been described in models and in clinical settings by the use of advanced MRI approaches [16,87,110,111,112]. As other authors have emphasized, glutamatergic neurons are largely present in the regions clinically affected by bilirubin and studied in this work. Among glutamatergic neurons, a part of them is positive for the parvalbumin marker. The marker identifies the neurons whose activity is calcium-dependent, a type of neuron that has been associated with cognitive and behavioral processes [16,113,114]. Noteworthy, the only gene permanently altered in all the regions under investigation in this work is *Grm1*, a metabotropic glutamate receptor involved in neurite outgrowth and post-synaptic activity [28,38,40,62,63,115]. Those also altered are *Cacng8* (promoting the targeting to the cell membrane and synapses of AMPA-selective glutamate receptors and modulating their gating properties [28,38]), and Cacna2d4 (a voltage-gated calcium channel; with the α2δ subunits as important regulators of synapse formation [28,38,116]). Notably, glutamate and calcium are interconnected in glutamate neurotoxicity, a well-known damaging mechanism in bilirubin neurotoxicity [16,23,116,117,118,119,120,121,122], in which Tnr, another gene we found to be modulated, may play a role, being involved in astrocyte-mediated glutamate tissue homeostasis [68]. Moreover, it is interesting to note that the core of the IC is dominated by glutamatergic neurons, differently from its cortical area [123], and in the core but not the periphery of the IC, we noticed necrosis at P9, suggesting a possible link between the transcriptomic and histologic observations. Notably, recently we described how curcumin conferred protection against (among other mechanisms of damage) glutamate neurotoxicity in our Gunn rats [52]. As a result of the discoveries we reported in this work, curcumin protection in non-Cll regions should be evaluated. 

As a matter of curiosity, due to the use of MRI in the diagnosis and management of kernicterus, IC is also interesting for the significant increase of *Slc39a12* at P9, a Zn transporter suggested to be responsible for interferences in T1 MRI signals due to magnetic resonance imaging being sensitive to metal content in the tissues [74,75,76,77,78,79,80]. 

Additionally, bilirubin widely perturbed the physiological program of brain development, leading to what we described as a di-synchrony of gene expression between Ctrls and jj rats (by destructing the physiological clusters), and a flattening of the physiologic transcriptome profile (by subverting multiple negative correlations), suggesting a potential for the presence of abnormal brain functions in jj animals. As pointed out by Das et al. [111], Wisnowski et al. [23], and Amin et al. [88], we cannot consider each region independently because they are part of neuronal pathways, where the alterations present in one region will functionally affect the others connected. Thus, it is of final importance that the behavior tests we performed support the presence of long-lasting neurological deficits, confirming the genetic rather than the histologic data. Proteomic analysis is needed to better understand the biological outcome of bilirubin neurotoxicity. 

From a clinical point of view, icterus appears in the second to fourth days of life and may persist for 1 or 2 weeks in untreated infants [2,124,125]. If blood bilirubin reaches the threshold for treatment, PT or exchange transfusions are usually efficient in reducing bilirubin and avoiding neurotoxicity. Despite this, multiple pieces of evidence argue for the presence of neurologic and neuro-behavioral conditions (e.g., hyperactivity, attention deficits, cognitive deficits, schizophrenia, bipolar disorders, and autism) in adults that experienced neonatal hyperbilirubinemia (mild and severe treated hyperbilirubinemia) [88,126,127,128,129,130]. Among the genes that we reported as permanently altered, *Camlg*, *Grm1*, *Cacna2d4*, *Cacng8*, and *Slc39a12* are known in the literature for their potential involvement in the just mentioned conditions [74,75,78,131,132,133,134,135,136,137,138,139,140]. Thus, our findings might contribute to understanding the late neurologic effects of early exposure to bilirubin. Studies dedicated to the goal are dedicated. Up until now, the Gunn rat has been used only for studying schizophrenia, but the studies have been done only in Hip, in adult animals, and quite exclusively focusing on inflammation and microgliosis, so comparing the results is impossible [93,136]. 

Among the altered genes, we noticed *Tnr*, a constituent of peri-neuronal nets, acting in both a negative and positive manner on neuronal and neurite growth, synapses maintenance, oligodendrocyte adhesion, and differentiation [28,37,68,69]. Its loss has been suggested to lead to a non-progressive neurodevelopmental disorder with spasticity and transient opisthotonus [28,37,68,69,140]. Notably, opisthotonus is a landmark feature of chronic bilirubin encephalopathy [111,141,142], adding interest in going deeper with the investigations following the indication arising from this exploratory work. 

We are aware that most of the discussion is speculative because it is based only on transcriptomic data. For sure, proteomic (protein level) and immunologic (localization/morphology of the structure; counting of neuronal cell types) confirmation is needed to deeply understand how bilirubin perturbs the dynamics of brain development and the possible resilience of each area of the CNS. Nevertheless, the agreement with the literature we mentioned is reassuring for the hypotheses that we may formulate starting from the present data to address future studies. 

## 5. Conclusions

The transcriptomic profile of bilirubin on brain development from the early postnatal period until adult age we depicted not only corroborated the knowledge of the regional sensitivity to bilirubin neurotoxicity but also provided new knowledge on the impact of the pigment on brain development, individuating new potential molecular targets and mechanisms. The findings we described are well in agreement with the clinic, open a way for better deciphering the neurotoxic features of bilirubin, and deeply evaluate the efficacy of new therapeutic approaches against the acute and long-lasting sequel of bilirubin neurotoxicity.

## Data Availability

All the data is contained within the article or Supplementary Materials [Figure S1: Representative pictures of the fields used for morphometric analysis; Figure S2: Easy recap of the peak of mRNA expression in normobilirubinemic animals; Table S2: Details on the biological functions of the studied genes based on the literature].

## Supplementary Materials

The following supporting information can be downloaded at: https://www.mdpi.com/article/10.3390/biology12060834/s1, Table S1: primers definition; Table S2: Details on the biological functions of the studied genes based on the literature; Figure S1: Representative pictures of the fields used for morphometric analysis; Figure S2: Easy recap of the peak of mRNA expression in normobilirubinemic animals.
