# Supplementary material for: Bilirubin-Induced Transcriptomic Imprinting in Neonatal Hyperbilirubinemia
Source: Biology (Basel). 2023 Jun 8;12(6):834. doi: 10.3390/biology12060834 (PMC10295065; doi:10.3390/biology12060834)
Supplement: Supplementary file 1 [file biology-12-00834-s001.zip › biology-2409575-supplementary.pdf]

JP Llido et. Al, Supplementary Materials

Biology **2023**, *12*(6), 834; https://doi.org/10.3390/biology12060834

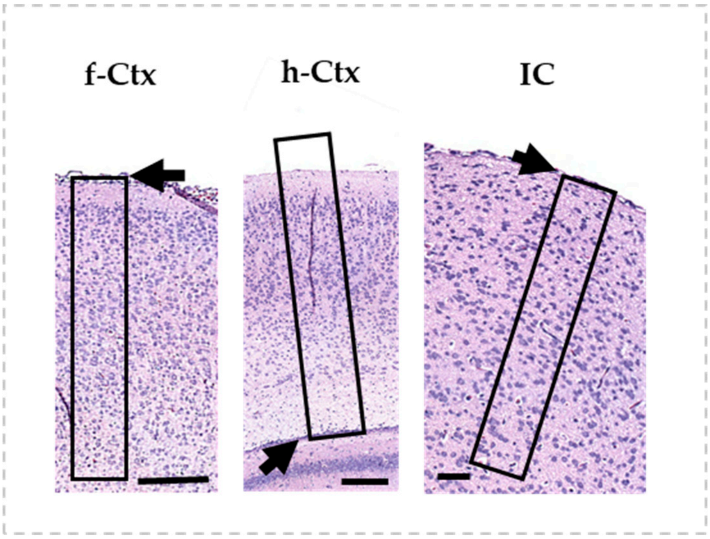

Figure S1: Representative pictures of the fields used for morphometric analysis. f-Ctx: frontal cortex; h-Ctx: parietal cortex; IC: inferior colliculi. Squares are representations of the fields used to quantify the number of the cells in each brain area of interest. Squares of fixed size have been used at each post-natal age to allow a proper comparison of the areas. Arrows indicate the reference point used to orienteering the fields.

Table S1. Primers definition. *Bmp5*: bone morphogenetic protein 5; *Cacna2d4*: calcium channel, voltage-dependent, alpha 2/delta subunit 4; *Cacng8*: calcium voltage-gated channel auxiliary subunit gamma 8; *Camlg*: calcium modulating ligand; *Casp6*: caspase 6; *Col4a3*: collagen 4a3; *Grm1*: glutamate metabotropic receptor 1; *Hyal4*: hyaluronidase 4; *Nduf7/8*: NADH: ubiquinone oxidoreductase core subunit 7/8; *Nstr1*: neurotensin receptor 1; *Pfkfb1*: 6-phosphofructo-2-kinase/fructose-2,6-biphosphatase 1; *Ptn*: pleiotrophin; *Scg2*: secretogranin II; *Slc39a12*: solute carrier family 39 member 12; *Slit3*: slit guidance ligand 3; *Thbs2*: thrombospondin-2; *Tnr*: tenascin R; *Hprt1*: hypoxanthine phosphor-ribosyl-transferase 1.

Primers Definition	NM	Forward	Reverse	Efficency
Bmp5	NM_001108168	CAGTCTACACGATACCAAT	GTAATGCCTTCTCTGATGA	70.5
Cacna2d4	NM_001191751	CGTCTATATGTCCGAAGC	AATACTGCCAGGTCAATG	69.5
Cacng8	NM_080696	ATCATTGAAACGCTGGAAT	CCTATGGTGGTCAGTAGT	90.1
Camlg	NM_053334	TTGTCTATATTCGCTCCATT	CACTGTCGTCTTTACCTT	70.3
Casp6	NM_031775	ACAGATGGCTTCTACAGA	AGTTCCTCTCCTCTTGTG	102.2
Col4a3	NM_001135759	TCACCACAATGCCATTCTTA	CGACAGCCAGTATGAATAGT	94.5
Grm1	NM_017011	TATATCATTCTGGCTGGTATT	AGGATGTGGTAGTAGGTT	93.3
Hyal4	NM_001100780	CTCGCCGTCTTCACTATC	AACTTACACTACCTCTTCTTCAA	98.4
Nduf7	NM_001008525	GCTACTACCACTACTCCTACT	CAGCCTGGCACATAGATG	86.9
Nduf8	NM_001106360	CAAGAAGTATAATATGCGTGTG	ATCGGGTAGTCACCATAC	88.4
Ntsr1	NM_001108967	TCGGATGAACAGTGGACTA	GTAGAAGAGAGCGTTGGTTAG	99.5
Pfkfb1	NM_012621	CACGCTATCTCAACTGGAT	CTGTAACTCACTGCCTCTC	65.5
Ptn	NM_017066	TGAAGACTCAGAGATGTAAGA	AAGCCTGGAACTGGTATT	58.1
Scg2	NM_022669	TAGAGCCAACCAGATTCC	TCCTTATCATTCAGATTGTCATAG	88.6
Slc39a12	NM_001106124	CTCTCCTCCTCCTCTATTAC	CTCATATTCTCTGTTGCTCAT	83.1
Slit3	NM_031321	TACGCCTAGAACAGAACT	TCTTGCTGATGTCTATTCG	76.7
Thbs2	NM_001169138	TGATAACAATGAGGACATAGATG	CTGGTTGGAGTTGGAGAT	78.3
Tnr	NM_013045	TCCAACTACCAAGACTACC	TTCATTACCGCAGATATTCC	86.9
Hprt1	NM_012583.2	AGACTGAAGAGCTACTGTAATGAC	GGCTGTACTGCTTGACCAAG	94.9

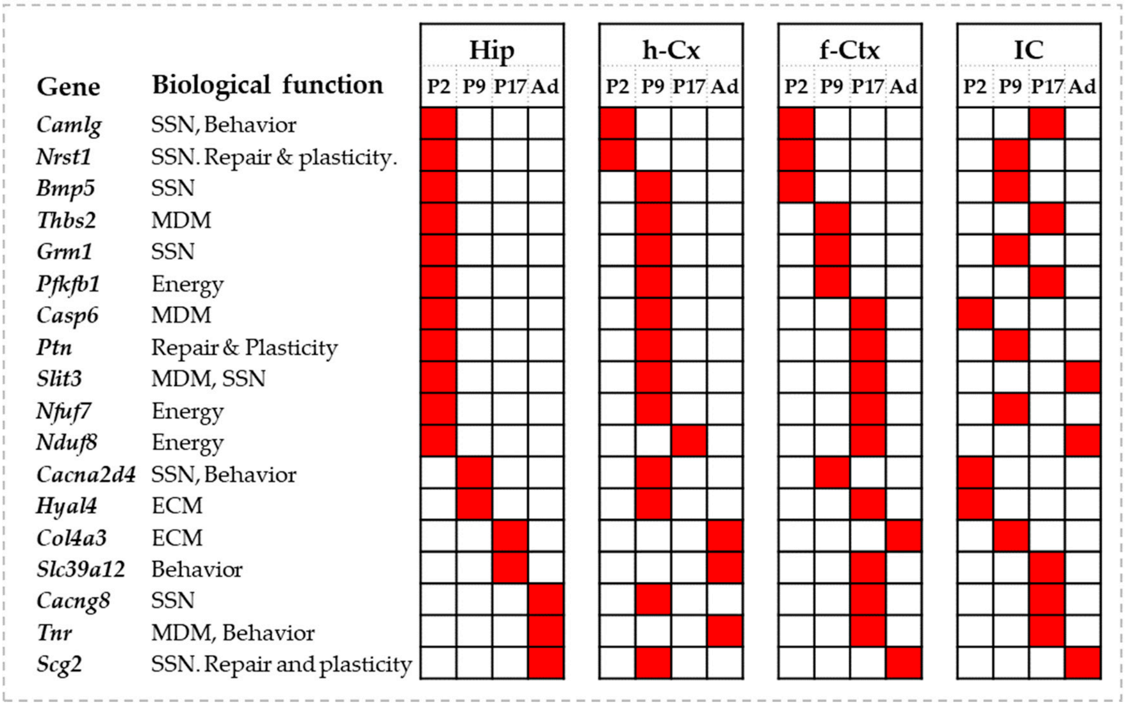

Figure S2: Easy recap of the peak of mRNA expression in normobilirubinemic animals.
Red squaes indicate the post-natal age and region of the brain where each gene presented the maximal expression. SSN: Synaptogenesis, synaptic activity, neuronal circuits establishment; B: behaviour; RP: repair and plasicity; MDM: Migration, differentiation, morphogenesis, E: energy. Hip: hippocampus; h-Ctx: parietal cerebral cortex; f-Ctx: frontal cerebral cortex; IC: inferior colliculi. Bmp5: bone morphogenetic protein; Camlg: calcium modulating ligand; Casp6: caspase 6; Col4a3: collagenase 4a3; Cacna2d4: calcium voltage-dependent calcium channel complex alpha-2/delta subunit family; Cacng8: calcium voltage-gated channel auxiliary subunit gamma 8; Grm1: glutamate metabotropic receptor 1; Hyal4: hyaluronic acid 4; Nstr1: neurotensin receptor 1; Nduf7/8: NADH: ubiquinone oxidoreductase (complex I) subunit A7/A8; Slit3: slit guidance ligand 3; Scg2: secretogranin II; Slc39a12: solute carrier family 39 member 12; Tnr: tenascin R; Thbs2: thrombospondin 2; Ptn: pleiotrophin; Pfkfb1: 6-phosphofructo-2-kinase/fructose-2,6-biphosphatase 1.

Table S2: Details on the biological functions of the studied genes based on the literature. Bmp5: Bone Morphogenetic Protein 5; Cacna2d4: calcium channel, voltage-dependent, alpha 2/delta subunit 4; Cacng8: Calcium Voltage-Gated Channel Auxiliary Subunit Gamma 8; Camlg: Calcium Modulating Ligand; Casp6: Caspase 6; Col4a3: Collagen 4a3; Grm1: Glutamate Metabotropic Receptor 1; Hyal4: Hyaluronidase 4; Nduf7: NADH:Ubiquinone Oxidoreductase Core Subunit S7; Nduf8: NADH:Ubiquinone Oxidoreductase Core Subunit S8; Nstr1: Neurotensin Receptor 1; Pfkfb1: 6-Phosphofructo-2-Kinase/Fructose-2,6-Biphosphatase 1; Ptn: Pleiotrophin; Scg2: Secretogranin II; Slc39a12: Solute Carrier Family 39 Member 12; Slit3: Slit Guidance Ligand 3; Thbs2: Thrombospondin-2; Tnr: Tenascin R.

Biological Process	Gene	Short Description	Reference
**Migration, Differentiation, Morphogenesis**	*Slit3*	Slit acts as chemo-repellents in axonal guidance.	*37.*
	Tnr2	Constituent of peri-neuronal nets. Acts in both negative and positive way on neurons and neurites growth, synapses maintenance, oligodendrocytes adhesion and differentiation. Regulates astrocyte glutamate up-take in adult brain. Regulates Na channel function. Enriched in oligodendrocyte precursor cells.	*37;* 28; 68; *69.*
	Casp6	Apoptosis, important on achieving the final architecture of the mature, functional CNS.	64; 65; 66; 67.
	Thbs2	Compartmentalization of the extracellular matrix. Mediates cell-cell and cell-matrix interactions in angiogenesis, inflammation, osteogenesis, cell proliferation, and apoptosis. Induces presynaptic differentiation in the CNS. Promoting the formation of new synapses in brain development and repair. Involved in BBB repair.	*37;* 84; 85; 86.
**Extracellular Matrix (ECM)**	*Col4a3*	Collagen IV is a structural component of basal laminae and found in neurogenic niche.	*37;* 31.
	Hyal4	One of the major component of the CNS's ECM. Forms the backbone of the ECM in the brain. Limits lateral diffusion of AMPARs; promotes activity of L-type Ca^{2+} channels.	37; 29; 33.
**Synaptogenesis, Synaptic activity, Neuronal circuits establishment**	*Thbs2*	Compartmentalization of the extracellular matrix. Mediates cell-cell and cell-matrix interactions in angiogenesis, inflammation, osteogenesis, cell proliferation, and apoptosis. Promoting the formation of new synapses in brain development and repair. Involved in BBB repair.	37; 84; 85; 86.
	Cacna2d4	Voltage-gated calcium channel; with the $\alpha 2\delta$ subunits as important regulators of synapse formation.	28; 38.
	Cacng8	Promotes the targeting to the cell membrane and synapses of AMPA-selective glutamate receptors (AMPARs), and modulates their gating properties by slowing their rates of activation, deactivation and desensitization and by mediating their resensitization.	28; *38.*
	Bmp5	Involved in extension and survival of dendrites. Involved in stem cell neurogenesis both in embryonic life as well in the adult life (sub-ventricular zone SVZ).	*29; 33; 39.*
	Grm1	Metabotropic glutamate receptor, post-synaptic activity. Involved in stem cell neurogenesis in SVZ.	28; *62;* 63; *38.*
	Ntsr1	Synaptogenesis, plasticity, neuronal circuity.	28; 58; 59; 60; 61.

	Camlg	Regulates Membrane Trafficking of Postsynaptic GABAA Receptors. Involved in many signaling cascades.	*62; 38.*
	Slit3	Acts as chemo-repellents in axonal guidance.	*37.*
	Scg2	Synaptogenesis, neuronal circuits formation, plasticity & repair. Involved in neurotransmission and paracrine regulation of central and peripheral actions of the nervous and neuroendocrine systems.	38.
**Repair Plasticity**	*Bmp5*	Involved in extension and survival of dendrites. Involved in stem cell neurogenesis both in embryonic life as well in the adult life (sub-ventricular zone: SVZ).	29; 33; 39.
	Ntsr1	Synaptogenesis, plasticity, neuronal circuity.	28; 58; 59; 60; 61.
	Ptn	Repair and plasticity. Secreted heparin-binding growth factor, and pro-inflammatory cytokine, acting as a neuromodulator, suggested to be involved in axonal outgrowth, and in capability of injured neurons to regenerate.	41; 81; 83; 82.
	Scg2	Synaptogenesis, neuronal circuits formation, plasticity & repair. Involved in neurotransmission and paracrine regulation of central and peripheral actions of the nervous and neuroendocrine systems	38.
**Energy**	*Nduf7/8*	Core subunit of the mitochondrial membrane respiratory chain NADH dehydrogenase (Complex I) that is believed to belong to the minimal assembly required for catalysis. Affected in mitochondrial dysfunction, active in ER stress. Suggested to be essential to neuronal adaptation to injury.	28; *70;* 71; 72.
	Pfkfb1	Catalyzes both the synthesis (glycolysis) and degradation (gluconeogenesis) of fructose-2,6-biphosphate.	28; *73.*
**Behavior**	*Camlg*	It is thought to be an important mediator of learning and memory.	*137.*
	Grm1	Associated with many disease states, including schizophrenia, bipolar disorder, depression.	*139; 138.*
	Slc39a12	Zn transporter, necessary for neurite extension, and cellular respiration. It has been suggested to be involved in schizophrenia, possibly autism. Slc39a12 genes polymorphisms have been linked with abnormal T1 signals in MRI, being magnetic resonance imaging sensitive to metal content in the tissues.	74; 75; 76; 78.
	Cacng8	Attention-deficit hyperactivity disorder (hyperactivity, impulsivity, anxiety, impaired cognition, and memory deficits.	131; 132; 133; 134.
	Cacna2d4	Associated with bipolar-disorders.	135.
	Tnr	Loss of TNR causes a non-progressive neurodevelopmental disorder with spasticity and transient opisthotonus.	140.

References

28. Mody, M.; Cao, Y.; Cui, Z.; Tay, K.-Y.; Shyong, A.; Shimizu, E.; Pham, K.; Schultz, P.; Welsh, D.; Tsien, J. Z. Genome-Wide Gene Expression Profiles of the Developing Mouse Hippocampus. *Proc. Natl. Acad. Sci.* **2001**, *98* (15), 8862–8867. https://doi.org/10.1073/pnas.141244998.

29. Urbán, N.; Guillemot, F. Neurogenesis in the Embryonic and Adult Brain: Same Regulators, Different Roles. *Front. Cell. Neurosci.* **2014**, *8*. https://doi.org/10.3389/fncel.2014.00396.

31. Moeendarbary, E.; Weber, I. P.; Sheridan, G. K.; Koser, D. E.; Soleman, S.; Haenzi, B.; Bradbury, E. J.; Fawcett, J.; Franze, K. The Soft Mechanical Signature of Glial Scars in the Central Nervous System. *Nat. Commun.* **2017**, *8*, 14787. https://doi.org/10.1038/ncomms14787.

33. Miller, J. A.; Nathanson, J.; Franjic, D.; Shim, S.; Dalley, R. A.; Shapouri, S.; Smith, K. A.; Sunkin, S. M.; Bernard, A.; Bennett, J. L.; Lee, C.-K.; Hawrylycz, M. J.; Jones, A. R.; Amaral, D. G.; Šestan, N.; Gage, F. H.; Lein, E. S. Conserved Molecular Signatures of Neurogenesis in the Hippocampal Subgranular Zone of Rodents and Primates. *Dev. Camb. Engl.* **2013**, *140* (22), 4633–4644. https://doi.org/10.1242/dev.097212.

37 Dityatev, A.; Seidenbecher, C. I.; Schachner, M. Compartmentalization from the Outside: The Extracellular Matrix and Functional Microdomains in the Brain. *Trends Neurosci.* **2010**, *33* (11), 503–512. https://doi.org/10.1016/j.tins.2010.08.003.

38. Cho, Y.; Gong, T.-W. L.; Stöver, T.; Lomax, M. I.; Altschuler, R. A. Gene Expression Profiles of the Rat Cochlea, Cochlear Nucleus, and Inferior Colliculus. *J. Assoc. Res. Otolaryngol. JARO* **2002**, *3* (1), 54–67. https://doi.org/10.1007/s101620010042.

39. Schubert, D.; Martens, G. J. M.; Kolk, S. M. Molecular Underpinnings of Prefrontal Cortex Development in Rodents Provide Insights into the Etiology of Neurodevelopmental Disorders. *Mol. Psychiatry* **2015**, *20* (7), 795–809. https://doi.org/10.1038/mp.2014.147.

40. Vallès, A.; Boender, A. J.; Gijsbers, S.; Haast, R. A. M.; Martens, G. J. M.; de Weerd, P. Genomewide Analysis of Rat Barrel Cortex Reveals Time- and Layer-Specific MRNA Expression Changes Related to Experience-Dependent Plasticity. *J. Neurosci. Off. J. Soc. Neurosci.* **2011**, *31* (16), 6140–6158. https://doi.org/10.1523/JNEUROSCI.6514-10.2011.

41. Decourt, B.; Bouleau, Y.; Dulon, D.; Hafidi, A. Identification of Differentially Expressed Genes in the Developing Mouse Inferior Colliculus. *Dev. Brain Res.* **2005**, *159* (1), 29–35. https://doi.org/10.1016/j.devbrainres.2005.06.010.

58. Boules, M.; Li, Z.; Smith, K.; Fredrickson, P.; Richelson, E. Diverse Roles of Neurotensin Agonists in the Central Nervous System. *Front. Endocrinol.* **2013**, *4*. https://doi.org/10.3389/fendo.2013.00036.

59. St-Gelais, F.; Jomphe, C.; Trudeau, L.-É. The Role of Neurotensin in Central Nervous System Pathophysiology: What Is the Evidence? *J Psychiatry Neurosci* 17.

60. Xiao, Z.; Cilz, N. I.; Kurada, L.; Hu, B.; Yang, C.; Wada, E.; Combs, C. K.; Porter, J. E.; Lesage, F.; Lei, S. Activation of Neurotensin Receptor 1 Facilitates Neuronal Excitability and Spatial Learning and Memory in the Entorhinal Cortex: Beneficial Actions in an Alzheimer's Disease Model. *J. Neurosci.* **2014**, *34* (20), 7027–7042. https://doi.org/10.1523/JNEUROSCI.0408-14.2014.

61. Woodworth, H. L.; Batchelor, H. M.; Beekly, B. G.; Bugescu, R.; Brown, J. A.; Kurt, G.; Fuller, P. M.; Leinninger, G. M. Neurotensin Receptor-1 Identifies a Subset of Ventral Tegmental Dopamine Neurons That Coordinates Energy Balance. *Cell Rep.* **2017**, *20* (8), 1881–1892. https://doi.org/10.1016/j.celrep.2017.08.001.

62. Holt, A. G.; Asako, M.; Lomax, C. A.; MacDonald, J. W.; Tong, L.; Lomax, M. I.; Altschuler, R. A. Deafness-Related Plasticity in the Inferior Colliculus: Gene Expression Profiling Following Removal of Peripheral Activity. *J. Neurochem.* **2005**, *93* (5), 1069–1086. https://doi.org/10.1111/j.1471-4159.2005.03090.x.

63. Hagihara, H.; Ohira, K.; Toyama, K.; Miyakawa, T. Expression of the AMPA Receptor Subunits GluR1 and GluR2 Is Associated with Granule Cell Maturation in the Dentate Gyrus. *Front. Neurosci.* **2011**, *5*. https://doi.org/10.3389/fnins.2011.00100.

64. Uribe, V.; Wong, B. K. Y.; Graham, R. K.; Cusack, C. L.; Skotte, N. H.; Pouladi, M. A.; Xie, Y.; Feinberg, K.; Ou, Y.; Ouyang, Y.; Deng, Y.; Franciosi, S.; Bissada, N.; Spreeuw, A.; Zhang, W.; Ehrnhoefer, D. E.; Vaid, K.; Miller, F. D.; Deshmukh, M.; Howland, D.; Hayden, M. R. Rescue from Excitotoxicity and Axonal Degeneration Accompanied by Age-Dependent Behavioral and Neuroanatomical Alterations in Caspase-6-Deficient Mice. *Hum. Mol. Genet.* **2012**, *21* (9), 1954–1967. https://doi.org/10.1093/hmg/dds005.

65. Nikolić, M.; Gardner, H. A. R.; Tucker, K. L. Postnatal Neuronal Apoptosis in the Cerebral Cortex: Physiological and Pathophysiological Mechanisms. *Neuroscience* **2013**, *254*, 369–378. https://doi.org/10.1016/j.neuroscience.2013.09.035.
66. Pfisterer, U.; Khodosevich, K. Neuronal Survival in the Brain: Neuron Type-Specific Mechanisms. *Cell Death Dis.* **2017**, *8* (3), e2643–e2643. https://doi.org/10.1038/cddis.2017.64.
67. Bandeira, F.; Lent, R.; Herculano-Houzel, S. Changing Numbers of Neuronal and Non-Neuronal Cells Underlie Postnatal Brain Growth in the Rat. *Proc. Natl. Acad. Sci.* **2009**, *106* (33), 14108–14113. https://doi.org/10.1073/pnas.0804650106.
68. Okuda, H.; Tatsumi, K.; Morita, S.; Shibukawa, Y.; Korekane, H.; Horii-Hayashi, N.; Wada, Y.; Taniguchi, N.; Wanaka, A. Chondroitin Sulfate Proteoglycan Tenascin-R Regulates Glutamate Uptake by Adult Brain Astrocytes*. *J. Biol. Chem.* **2014**, *289* (5), 2620–2631. https://doi.org/10.1074/jbc.M113.504787.
69. Gottschling, C.; Wegrzyn, D.; Denecke, B.; Faissner, A. Elimination of the Four Extracellular Matrix Molecules Tenascin-C, Tenascin-R, Brevican and Neurocan Alters the Ratio of Excitatory and Inhibitory Synapses. *Sci. Rep.* **2019**, *9* (1), 13939. https://doi.org/10.1038/s41598-019-50404-9.
70. Eckert, A.; Schmitt, K.; Götz, J. Mitochondrial Dysfunction - the Beginning of the End in Alzheimer's Disease? Separate and Synergistic Modes of Tau and Amyloid-β Toxicity. *Alzheimers Res. Ther.* **2011**, *3* (2), 15. https://doi.org/10.1186/alzrt74.
71. Foti, S. C.; Hargreaves, I.; Carrington, S.; Kiely, A. P.; Houlden, H.; Holton, J. L. Cerebral Mitochondrial Electron Transport Chain Dysfunction in Multiple System Atrophy and Parkinson's Disease. *Sci. Rep.* **2019**, *9* (1), 6559. https://doi.org/10.1038/s41598-019-42902-7.
72. Knowlton, W. M.; Hubert, T.; Wu, Z.; Chisholm, A. D.; Jin, Y. A Select Subset of Electron Transport Chain Genes Associated with Optic Atrophy Link Mitochondria to Axon Regeneration in Caenorhabditis Elegans. *Front. Neurosci.* **2017**, *11*.
73. Stansberg, C.; Ersland, K. M.; van der Valk, P.; Steen, V. M. Gene Expression in the Rat Brain: High Similarity but Unique Differences between Frontomedial-, Temporal- and Occipital Cortex. *BMC Neurosci.* **2011**, *12*, 15. https://doi.org/10.1186/1471-2202-12-15.
74. Scarr, E.; Udawela, M.; Greenough, M. A.; Neo, J.; Suk Seo, M.; Money, T. T.; Upadhyay, A.; Bush, A. I.; Everall, I. P.; Thomas, E. A.; Dean, B. Increased Cortical Expression of the Zinc Transporter SLC39A12 Suggests a Breakdown in Zinc Cellular Homeostasis as Part of the Pathophysiology of Schizophrenia. *Npj Schizophr.* **2016**, *2* (1), 1–7. https://doi.org/10.1038/npjschz.2016.2.
75. Davis, D. N.; Strong, M. D.; Chambers, E.; Hart, M. D.; Bettaieb, A.; Clarke, S. L.; Smith, B. J.; Stoecker, B. J.; Lucas, E. A.; Lin, D.; Chowanadisai, W. A Role for Zinc Transporter Gene SLC39A12 in the Nervous System and Beyond. *Gene* **2021**, *799*, 145824. https://doi.org/10.1016/j.gene.2021.145824.
76. Strong.; Md, H.; Tz, T.; Ba, O.; L, W.; Mr, N.; Wt, A.; Hj, H.; Pr, H.; A, B.; Sl, C.; Bj, S.; Bj, S.; Ea, L.; D, L.; W, C. Role of Zinc Transporter ZIP12 in Susceptibility-Weighted Brain Magnetic Resonance Imaging (MRI) Phenotypes and Mitochondrial Function. *FASEB J. Off. Publ. Fed. Am. Soc. Exp. Biol.* **2020**, *34* (9). https://doi.org/10.1096/fj.202000772R.
84. Zhou, H.-J.; Zhang, H.-N.; Tang, T.; Zhong, J.-H.; Qi, Y.; Luo, J.-K.; Lin, Y.; Yang, Q.-D.; Li, X.-Q. Alteration of Thrombospondin-1 and -2 in Rat Brains Following Experimental Intracerebral Hemorrhage: Laboratory Investigation. *J. Neurosurg.* **2010**, *113* (4), 820–825. https://doi.org/10.3171/2010.1.JNS09637.
78. Bly, M. Examination of the Zinc Transporter Gene, SLC39A12. *Schizophr. Res.* **2006**, *81* (2–3), 321–322. https://doi.org/10.1016/j.schres.2005.07.039.
81. González-Castillo, C.; Ortuño-Sahagún, D.; Guzmán-Brambila, C.; Pallàs, M.; Rojas-Mayorquín, A. E. Pleiotrophin as a Central Nervous System Neuromodulator, Evidences from the Hippocampus. *Front. Cell. Neurosci.* **2015**, *8*.
82. Wanaka, A.; Carroll, S. L.; Milbrandt, J. Developmentally Regulated Expression of Pleiotrophin, a Novel Heparin Binding Growth Factor, in the Nervous System of the Rat. *Dev. Brain Res.* **1993**, *72* (1), 133–144. https://doi.org/10.1016/0165-3806(93)90166-8.
83. Fernández-Calle, R.; Vicente-Rodríguez, M.; Gramage, E.; Pita, J.; Pérez-García, C.; Ferrer-Alcón, M.; Uribarri, M.; Ramos, M. P.; Herradón, G. Pleiotrophin Regulates Microglia-Mediated Neuroinflammation. *J. Neuroinflammation* **2017**, *14* (1), 46. https://doi.org/10.1186/s12974-017-0823-8.

84. Zhou, H.-J.; Zhang, H.-N. Alteration of Thrombospondin-1 and -2 in Rat Brains Following Experimental Intracerebral 955 Hemorrhage: Laboratory Investigation. J. Neurosurg. 2010, 113 (4), 820–825. https://doi.org/10.3171/2010.1.JNS09637
85. Iruela-Arispe, M. L.; Liska, D. J.; Sage, E. H.; Bornstein, P. Differential Expression of Thrombospondin 1, 2, and 3 during Murine Development. *Dev. Dyn.* **1993**, *197* (1), 40–56. https://doi.org/10.1002/aja.1001970105.
86. Wang, S.; Hu, T.; Wang, Z.; Li, N.; Zhou, L.; Liao, L.; Wang, M.; Liao, L.; Wang, H.; Zeng, L.; Fan, C.; Zhou, H.; Xiong, K.; Huang, J.; Chen, D. Macroglia-Derived Thrombospondin 2 Regulates Alterations of Presynaptic Proteins of Retinal Neurons Following Elevated Hydrostatic Pressure. *PLOS ONE* **2017**, *12* (9), e0185388. https://doi.org/10.1371/journal.pone.0185388.
131. Bai, W.-J.; Luo, X.-G.; Jin, B.-H.; Zhu, K.-S.; Guo, W.-Y.; Zhu, X.-Q.; Qin, X.; Yang, Z.-X.; Zhao, J.-J.; Chen, S.-R.; Wang, R.; Hao, J.; Wang, F.; Shi, Y. S.; Kong, D.-Z.; Zhang, W. Deficiency of Transmembrane AMPA Receptor Regulatory Protein γ-8 Leads to Attention-Deficit Hyperactivity Disorder-like Behavior in Mice. *Zool. Res.* **2022**, *43* (5), 851–870. https://doi.org/10.24272/j.issn.2095-8137.2022.122.
132. Arnsten, A. F. T.; Pliszka, S. R. Catecholamine Influences on Prefrontal Cortical Function: Relevance to Treatment of Attention Deficit/Hyperactivity Disorder and Related Disorders. *Pharmacol. Biochem. Behav.* **2011**, *99* (2), 211–216. https://doi.org/10.1016/j.pbb.2011.01.020.
133. Biederman, J. Attention-Deficit/Hyperactivity Disorder: A Selective Overview. *Biol. Psychiatry* **2005**, *57* (11), 1215–1220. https://doi.org/10.1016/j.biopsych.2004.10.020.
134. Sharma, A.; Couture, J. A Review of the Pathophysiology, Etiology, and Treatment of Attention-Deficit Hyperactivity Disorder (ADHD). *Ann. Pharmacother.* **2014**, *48* (2), 209–225. https://doi.org/10.1177/1060028013510699.
135. Van Den Bossche, M. J.; Strazisar, M.; De Bruyne, S.; Bervoets, C.; Lenaerts, A.-S.; De Zutter, S.; Nordin, A.; Norrback, K.-F.; Goossens, D.; De Rijk, P.; Green, E. K.; Grozeva, D.; Mendlewicz, J.; Craddock, N.; Sabbe, B. G.; Adolfsson, R.; Souery, D.; Del-Favero, J. Identification of a CACNA2D4 Deletion in Late Onset Bipolar Disorder Patients and Implications for the Involvement of Voltage-Dependent Calcium Channels in Psychiatric Disorders. *Am. J. Med. Genet. Part B Neuropsychiatr. Genet. Off. Publ. Int. Soc. Psychiatr. Genet.* **2012**, *159B* (4), 465–475. https://doi.org/10.1002/ajmg.b.32053.
137. Wilson, M. P.; Durin, Z.; Unal, Ö.; Ng, B. G.; Marrecau, T.; Keldermans, L.; Souche, E.; Rymen, D.; Gündüz, M.; Köse, G.; Sturiale, L.; Garozzo, D.; Freeze, H. H.; Jaeken, J.; Foulquier, F.; Matthijs, G. CAMLG-CDG: A Novel Congenital Disorder of Glycosylation Linked to Defective Membrane Trafficking. *Hum. Mol. Genet.* **2022**, *31* (15), 2571–2581. https://doi.org/10.1093/hmg/ddac055.
138. Blacker, C. J.; Lewis, C. P.; Frye, M. A.; Veldic, M. Metabotropic Glutamate Receptors as Emerging Research Targets in Bipolar Disorder. *Psychiatry Res.* **2017**, *257*, 327–337. https://doi.org/10.1016/j.psychres.2017.07.059.
139. De Rosa, A.; Fontana, A.; Nuzzo, T.; Garofalo, M.; Di Maio, A.; Punzo, D.; Copetti, M.; Bertolino, A.; Errico, F.; Rampino, A.; de Bartolomeis, A.; Usiello, A. Machine Learning Algorithm Unveils Glutamatergic Alterations in the Post-Mortem Schizophrenia Brain. *Schizophrenia* **2022**, *8* (1), 1–16. https://doi.org/10.1038/s41537-022-00231-1.
140. Wagner, M.; Lévy, J.; Jung-Klawitter, S.; Bakhtiari, S.; Monteiro, F.; Maroofian, R.; Bierhals, T.; Hempel, M.; Elmaleh-Bergès, M.; Kitajima, J. P.; Kim, C. A.; Salomao, J. G.; Amor, D. J.; Cooper, M. S.; Perrin, L.; Pipiras, E.; Neu, A.; Doosti, M.; Karimiani, E. G.; Toosi, M. B.; Houlden, H.; Jin, S. C.; Si, Y. C.; Rodan, L. H.; Venselaar, H.; Kruer, M. C.; Kok, F.; Hoffmann, G. F.; Strom, T. M.; Wortmann, S. B.; Tabet, A.-C.; Opladen, T. Loss of TNR Causes a Nonprogressive Neurodevelopmental Disorder with Spasticity and Transient Opisthotonus. *Genet. Med.* **2020**, *22* (6), 1061–1068. https://doi.org/10.1038/s41436-020-0768-7.
